# Engineering the Iron Center: Hemin Nanozymes for Programmable Cancer Catalysis

**DOI:** 10.1021/acs.nanolett.6c00429

**Published:** 2026-05-06

**Authors:** Amir M. Alsharabasy, Godspower T. Isaac, Abhay Pandit

**Affiliations:** CÚRAM, Research Ireland Centre for Medical Devices, 8799 University of Galway, Galway H91 W2TY, Ireland

**Keywords:** hemin nanozymes, tumor microenvironment, chemodynamic therapy, redox modulation, cascade nanoreactors

## Abstract

The tumor microenvironment (TME) presents coupled barriers, including hypoxia, acidity, elevated hydrogen peroxide levels, and high glutathione levels, which limit the efficacy of conventional cancer therapies. Hemin, an iron protoporphyrin cofactor of hemoglobin, has emerged as a versatile catalytic node for engineered nanozymes, enabling programmable redox modulation within the TME. Through rational nanoengineering, hemin-based architectures integrate chemodynamic, photodynamic, ferroptotic, and metabolic pathways to amplify reactive oxygen species, recycle oxygen, deplete antioxidant defense, and induce iron-dependent cell death. This Review examines the developments in hemin nanotechnology, with an emphasis on precision assembly, material engineering, cascade nanoreactors, and biological modulation. We have distilled these developments into a rational design toolbox that guides scaffold selection, coordination tuning, spatial programming, and translational optimization, establishing principles for next-generation tumor-adaptive redox nanomaterials.

## Graphical abstract

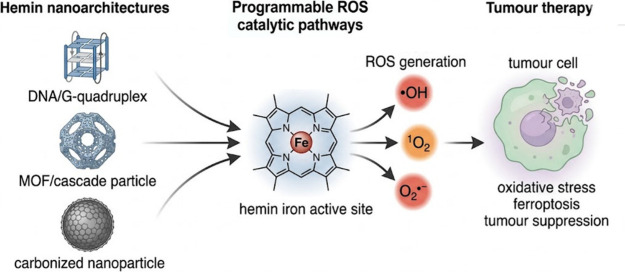

The tumor microenvironment (TME) presents multiple physicochemical barriers that limit the efficacy of conventional cancer therapies. Abnormal vasculature leads to hypoxia, elevated interstitial fluid pressure,^1^ and acidosis,^2^ accompanied by high hydrogen peroxide (H_2_O_2_) levels (∼0.1–1 mM) and intrinsically low catalase (CAT) activity.^3^ Impaired lymphatic drainage further restricts drug transport,^4^ exacerbating oxygen deprivation and acidic stress. In parallel, cancer cells maintain an elevated antioxidant capacity, characterized by high intracellular glutathione (GSH; ∼10 mM) levels and upregulated redox defense pathways that rapidly quench reactive oxygen species (ROS).^5,6^ Collectively, hypoxia, acidity, excess H_2_O_2_, and high GSH undermine oxygen-dependent therapies such as photodynamic therapy (PDT) and suppress ROS-mediated cytotoxicity, rendering many single-modality nanotherapy ineffective. To address these constraints, contemporary nanomedicine increasingly exploits, rather than avoids, the pathological features of the TME. Representative strategies include oxygen-generating or CAT-mimetic systems to alleviate hypoxia,^7^ Fenton-active iron- or copper-based catalysts that convert endogenous H_2_O_2_ into cytotoxic hydroxyl radicals (^•^OH),^8^ and GSH-consuming nanozymes that disable intracellular antioxidant buffering and sensitize tumors to oxidative damage.^6,9^ Multifunctional catalytic nanotherapeutics that integrate these capabilities can simultaneously enhance ROS production, improve intratumoral penetration, and amplify therapeutic efficacy.^3,9^

Porphyrin macrocycles, including chlorins and hemes, have long underpinned cancer theranostics owing to their photodynamic and imaging properties.^10^ Recent advances in nanoscale engineering have revitalized porphyrin-based platforms by enabling precise control over their photophysics, biodistribution, and catalytic activities. Examples include structure-dependent ^1^O_2_ generation in porphyrin assemblies;^11^ oxygen-reservoir porphyrin nanoparticles improving ^1^O_2_/hypoxia relief;^12^ renal-clearable porphyrinic MOF nanodots;^13^ and water-soluble porphyrin nanoparticles increasing ^1^O_2_/PDT.^14^

Iron protoporphyrin IX (hemin) is uniquely positioned at the interface between bioinorganic chemistry and nanomedicine, and its potential applications in nanomedicine are being explored. Its redox-active Fe­(III) center catalyzes H_2_O_2_ decomposition and radical generation, processes central to chemodynamic and photodynamic cancer therapy.^15^ When stabilized within nanoplatforms, such as carbon nanomaterials, metal–organic frameworks (MOFs), or DNA-based scaffolds, hemin functions as both a therapeutic agent and a catalytic nanozyme.^16^ For example, polymer-encapsulated carbonized hemin nanoparticles markedly amplify intratumoral oxidative stress by generating ^•^OH and singlet oxygen (^1^O_2_), alleviating hypoxia, depleting GSH, and enhancing PDT efficacy.^8^

Collectively, these advances demonstrate how nanoengineering has transformed hemin from a biological cofactor into a multifunctional redox nanozyme capable of addressing the interconnected TME barriers.^8^ This Review critically examines recent progress in hemin-integrated nanotechnologies for cancer therapy, organized into four thematic domains: (i) precision assembly and stimulus-responsive activation via DNA and supramolecular scaffolds; (ii) material engineering approaches that enhance photophysical and catalytic performance through carbonized and single-atom hemin architectures; (iii) cascade nanoreactors exploiting metabolic synergism; and (iv) biological modulation through ferroptosis and iron metabolism. Building on these advances, we consolidated emerging insights into a rational design toolbox, outlining practical guidelines for scaffold selection, coordination tuning, spatial programming, and translational optimization of next-generation hemin-based nanotherapeutics (Figure 1).

**Figure 1:**
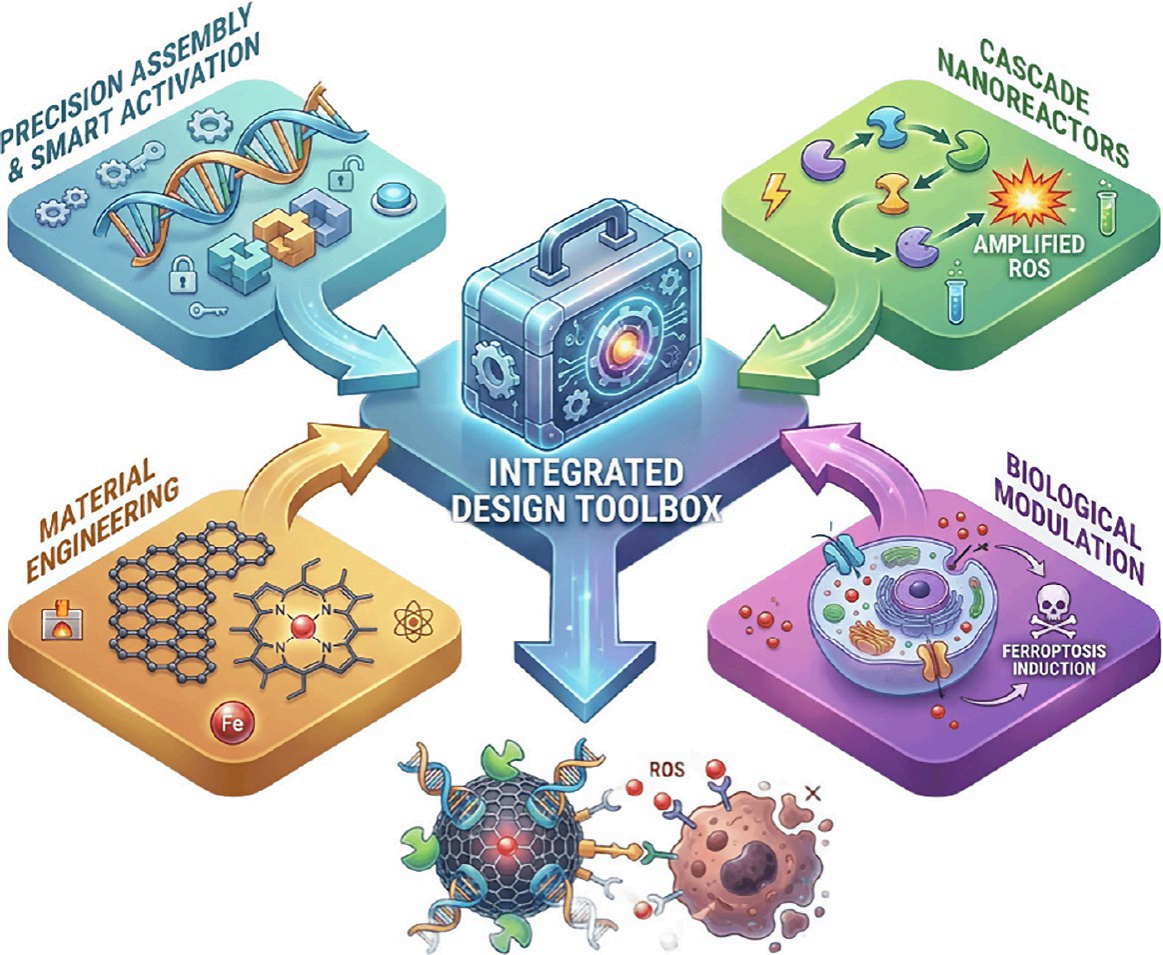
Integrated design toolbox and manuscript framework for hemin-based nanoarchitectures. *Created by illustrae.co.*

## Hemin as a Multifunctional Nanozyme: Chemical and Catalytic Foundations

### From Biological Cofactor to Engineered Nanozyme

Hemin (Fe-protoporphyrin IX chloride) is the catalytic core of heme proteins that mediate oxygen transport and redox homeostasis, including hemoglobin, myoglobin, peroxidases, and catalases.^16,15^ However, free hemin suffers from poor aqueous solubility^17^ and strong aggregation,^18^ limiting its direct biomedical use.^19,20^ These constraints have driven the development of engineered strategies to harness the catalytic potential of hemin. Although chemical derivatization of the porphyrin scaffold can modulate aggregation and activity, progress has been limited by synthetic complexity and restricted tunability.^18,21^ Consequently, embedding hemin within composite nanosystems has become the dominant biomedical strategy.

Nanoengineering stabilizes the Fe­(III) center and suppresses aggregation through confinement, coordination, or noncovalent interactions while imparting new physicochemical and biological functions. Hemin has been integrated across diverse platforms, including hydrogels, carbon nanomaterials, MOFs, and DNA G-quadruplex (G4) scaffolds,^16^ enabling precise control over accessibility and reactivity. Within these architectures, hemin is transformed from a passive cofactor to a multifunctional nanozyme that can operate under physiological conditions.

Engineered hemin nanozymes readily exhibit peroxidase (POD)-like activity; for example, hemin–graphene quantum dot assemblies display catalytic kinetics exceeding those of natural horseradish POD.^22^ In parallel, G-quadruplex–hemin complexes act as CAT mimics, efficiently decomposing H_2_O_2_ to molecular oxygen, a property critical for alleviating tumor hypoxia.^7^ Beyond POD and CAT functions, hemin nanozymes can oxidize thiols, mimicking glutathione peroxidase (GPx) or glutathione oxidase (OXD) activity.^23^ Systems such as Cu–hemin nanosheets exemplify this behavior by simultaneously depleting intracellular GSH and generating ROS, thereby sensitizing tumor cells to oxidative damage.^9^ Collectively, these advances establish hemin as a uniquely versatile catalytic node that integrates POD-, CAT-, and GPx-like activities at the nanoscale.

### Hemin-Mediated Chemodynamic, Photodynamic, Ferroptotic, and Metabolic Pathways

The therapeutic efficacy of hemin nanozymes arises from their redox-active iron center, which enables multiple interconnected tumor-destructive mechanisms. In chemodynamic therapy (CDT), the intracellular reduction of Fe­(III) to Fe­(II) allows Fenton or Fenton-like reactions with tumor-associated H_2_O_2_, generating highly cytotoxic ^•^OH that damage DNA, proteins, and lipid membranes.^8,24^ The acidic, H_2_O_2_-rich TME accelerates these reactions, conferring tumor-selective activity.^7^ Hemin also functions as a photosensitizer in PDT. Upon light irradiation, the excited porphyrin states transfer energy to oxygen to generate ^1^O_2_.^25^ Nanoscale confinement or coordination modulation can further enhance the photophysical efficiency.^7,21,25^ Importantly, CDT- and PDT-derived ROS act synergistically, with ^•^OH initiating rapid oxidative injury and ^1^O_2_ sustaining prolonged oxidative stress. This synergy is not accidental but arises from nano structural control over photophysical and catalytic pathways.^26^

Native hemin acts as a classical photosensitizer that predominantly generates singlet oxygen through a Type II photodynamic pathway.^7^ Upon light excitation, the porphyrin macrocycle transitions to an excited triplet state, which transfers energy to ground-state oxygen to form singlet oxygen (^1^O_2_).^27,28^ However, this pathway is inherently limited in solid tumors, where oxygen tensions are often below 2%, rapidly restricting ^1^O_2_ generation as oxygen becomes depleted.^26^

Material engineering strategies can reprogram this photophysical behavior. For example, solvothermal carbonization of hemin into polymer-encapsulated carbonized hemin nanoparticles creates conductive sp^2^-carbon frameworks containing dispersed iron catalytic sites.^8^ These carbon matrices act as efficient electron sinks, facilitating charge separation and suppressing electron–hole recombination after photoexcitation.^8^ As a result, the system shifts toward a Type I photodynamic mechanism dominated by electron transfer reactions rather than energy transfer. Electron transfer from the excited structure to surrounding substrates produces superoxide radicals (O_2_^•–^), which further convert into highly cytotoxic hydroxyl radicals (^•^OH).^28^ This nanoarchitectural transition therefore enables ROS generation even under hypoxic conditions.^5^ Importantly, residual porphyrin fragments may still generate singlet oxygen where oxygen is available, resulting in dual ROS production that amplifies oxidative stress within tumors.^8^

Beyond photophysics, the coordination environment of the central iron atom governs the ROS selectivity. Native hemin typically exhibits mixed peroxidase-like and catalase-like activity, where hydrogen peroxide can either generate cytotoxic radicals or be decomposed into oxygen and water.^29,30^ Nanostructural coordination engineering provides a strategy to control this balance.

For instance, axial chloride coordination modifies the electronic structure of the iron center, lowering the activation barrier for hydrogen peroxide decomposition into hydroxyl radicals and thereby enhancing peroxidase-like activity.^31,32^ In contrast, sulfur coordination environments, often introduced through cysteine-rich peptides or sulfide-based materials, stabilize high-spin iron states and suppress catalase pathways, forcing hydrogen peroxide conversion into hydroxyl radicals.^33^ Through such coordination tuning, nanoengineered hemin systems function as programmable catalytic centers that selectively promote ROS generation pathways relevant for tumor therapy, including ferroptosis-associated lipid peroxidation. Hemin-mediated redox chemistry also intersects with ferroptosis, an iron-dependent cell death pathway driven by lipid peroxide accumulation. By supplying redox-active iron while depleting intracellular GSH through GPx-like or GSH-OXD activity, hemin nanozymes disrupt GPx4-mediated lipid repair, triggering ferroptotic cell death.^7,9,24^ This mechanism is particularly valuable for targeting apoptosis-resistant tumors.

Finally, hemin nanozymes can also be integrated with metabolic starvation strategies. Cancer cells rely on aerobic glycolysis (the Warburg effect),^34^ and in cascade nanoreactors codelivering glucose oxidase (GOx), glucose consumption generates gluconic acid and H_2_O_2_.^35^ H_2_O_2_ fuels hemin-catalyzed ROS generation, and the resulting redox cascade simultaneously amplifies CDT/PDT activity and modulates tumor metabolism..^7,35^ By integrating ROS catalysis, ferroptosis induction, and metabolic modulation, hemin nanozymes function as multifunctional redox engines within the tumor microenvironment, motivating the design strategies discussed in the following sections.

## Advanced Hemin-Integrated Nanotechnologies as Cancer Therapeutics

Hemin-integrated nanotechnologies have matured from simple ROS-generating constructs into programmable, multimodule therapeutic systems that combine spatial control, catalytic amplification, and tumor-responsive activation. Here, we organize the field into four functional domains: (1) precision assembly via DNA and supramolecular scaffolds, (2) material engineering to tune photophysics and catalytic selectivity, (3) cascade nanoreactors that couple metabolic stress to amplified ROS, and (4) biological modulation leveraging ferroptosis and iron metabolism. Figure 2 summarizes how these modules converge into integrated reaction networks, and Table 1 consolidates the underlying reaction set.

**Figure 2:**
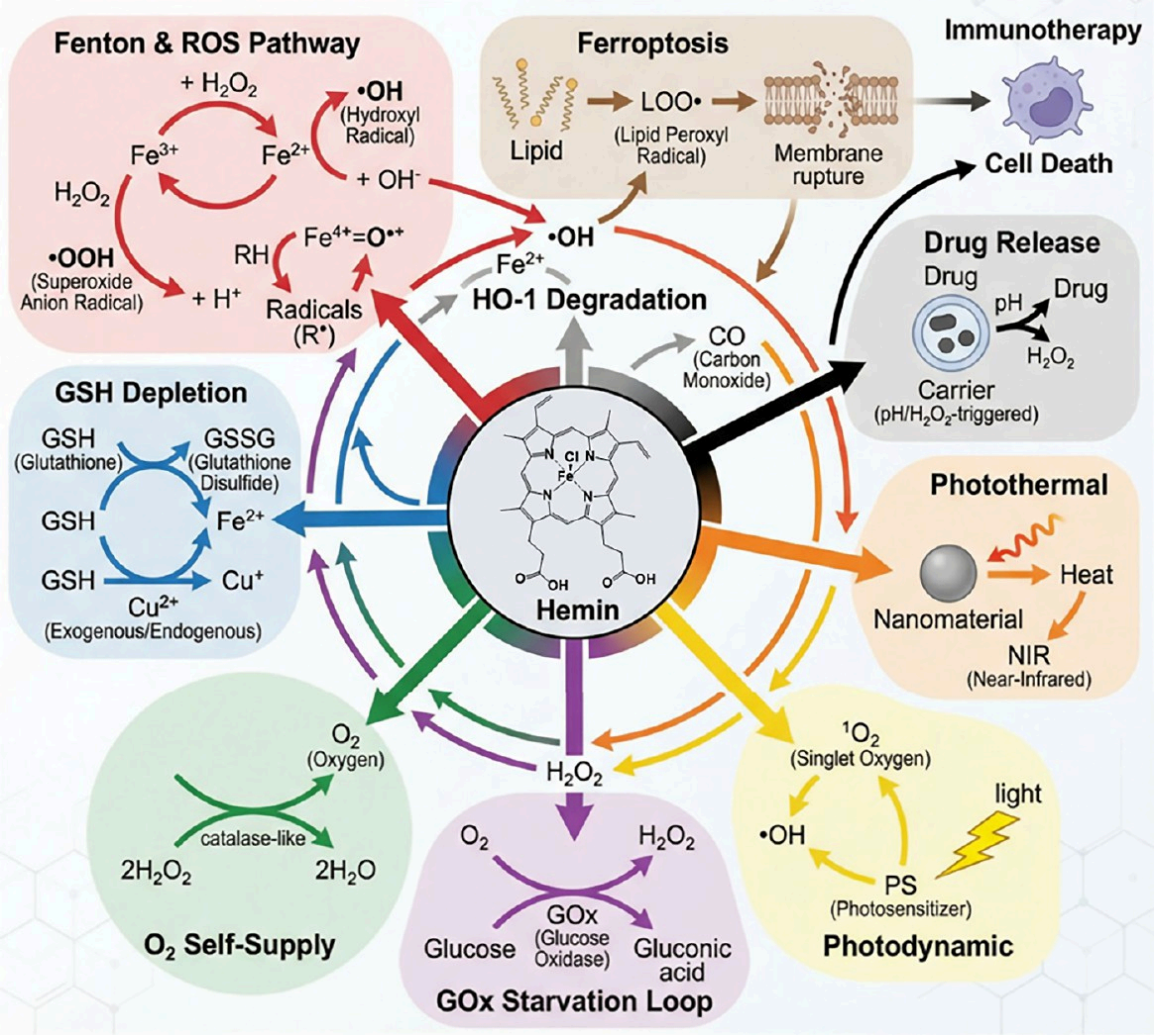
Hemin-orchestrated multimodal reaction network linking Fenton chemistry, GSH depletion, phototherapy, and ferroptosis. Each pathway and arrow in the schematic corresponds to the numbered reaction modules listed in Table 1 (Reactions 1–20). *Created by illustrae.co.*.

**Table 1:** Hemin-Centered Reaction Modules Governing ROS Generation, Redox Cycling, and Ferroptosis, Illustrated in Figure 2

Reaction module	Equation
Fenton and Fenton-like Reactions	1 ${\mathrm{Fe}}^{3+}\mathrm{-hemin}+H_{2}O_{2}\to {\mathrm{Fe}}^{4+}{\mathrm{=O}}^{•+}\mathrm{-hemin}+H_{2}O{\left(\mathrm{Peroxidase‐like}\right)}^{*}$
	2 ${\mathrm{Fe}}^{2+}+H_{2}O_{2}\to {\mathrm{Fe}}^{3+}+•\mathrm{OH}+{\mathrm{OH}}^{-}\left(\mathrm{Classical Fenton}\right)$
	3 ${\mathrm{Fe}}^{3+}+H_{2}O_{2}\to {\mathrm{Fe}}^{2+}+•\mathrm{OOH}+H^{+}\left(\mathrm{Photo‐Fenton with light}\right)$
	4 ${\mathrm{Fe}}^{4+}{\mathrm{=O}}^{•+}\mathrm{-hemin}+\mathrm{RH}\to {\mathrm{Fe}}^{3+}+R^{•}+H_{2}O\left(\mathrm{Radical generation}\right)$
GSH Depletion and Redox Cycling	5 $2\mathrm{GSH}+{\mathrm{Fe}}^{3+}\mathrm{-hemin}\to \mathrm{GSSG}+{\mathrm{Fe}}^{2+}\mathrm{-hemin}+2H^{+}$
	6 $2\mathrm{GSH}+2{\mathrm{Cu}}^{2+}\to \mathrm{GSSG}+2{\mathrm{Cu}}^{+}+2H^{+}$
Catalase-like Oxygen Generation	7 $2H_{2}O_{2}\to O_{2}+2H_{2}O\left(\mathrm{Hemin‐catalysed}\right)$
Glucose Oxidase (GOx) Cascade	8 $\mathrm{Glucose}+O_{2}+H_{2}O\to \mathrm{Gluconic acid}+H_{2}O_{2}$
Photothermal Therapy (PTT)	9 $\mathrm{Nanomaterial}\left(\mathrm{CuS},\mathrm{PDA},\mathrm{etc}.\right)+{h\nu }_{\mathrm{NIR}-\mathrm{II}}\to \mathrm{Heat}$
Photodynamic Therapy (PDT)	10 $\mathrm{PS}+h\nu \to {\mathrm{PS}}^{*}\left(\mathrm{excited state}\right)$
	11 ${\mathrm{PS}}^{*}{+}^{3}O_{2}\to \mathrm{PS}{+}^{1}O_{2}\left(\mathrm{Type II}\right)$
	12 ${\mathrm{PS}}^{*}+\mathrm{Substrate}\to {\mathrm{PS}}^{•-}+{\mathrm{Substrate}}^{•+}\to \mathrm{ROS}\left(\mathrm{Type I}\right)$
Lipid Peroxidation (Ferroptosis)	13 $\mathrm{LH}{+}^{•}\mathrm{OH}\to L^{•}+H_{2}O\left(\mathrm{Initiation}\right)$
	14 $L^{•}+H_{2}\to {\mathrm{LOO}}^{•}\left(\mathrm{Propagation}\right)$
	15 ${\mathrm{LOO}}^{•}+\mathrm{LH}\to \mathrm{LOOH}+L^{•}\left(\mathrm{Chain reaction}\right)$
	16 $\mathrm{LOOH}+{\mathrm{Fe}}^{2+}\to {\mathrm{LO}}^{•}+{\mathrm{OH}}^{-}+{\mathrm{Fe}}^{3+}\left(\mathrm{Fenton‐like on lipids}\right)$
Hemin Degradation (HO-1 Pathway)	17 $\mathrm{Hemin}+\mathrm{HO‐1}+O_{2}+\mathrm{NADPH}\to {\mathrm{Fe}}^{2+}+\mathrm{Biliverdin}+\mathrm{CO}+{\mathrm{NADP}}^{+}$
pH-Triggered Release	18 ${\mathrm{CaCO}}_{3}+2H^{+}\to {\mathrm{Ca}}^{2+}+{\mathrm{CO}}_{2}+H_{2}O\left(\mathrm{Calcium release}\right)$
β-Lapachone NQO1 Activation	19 β‐Lap+NQO1/NAD(P)H→Semiquinone
	20 $\mathrm{Semiquinone}+O_{2}\to \beta \mathrm{‐Lap}+{O_{2}}^{•-}\left(\to H_{2}O_{2}\;\mathrm{via dismutation}\right)$

### Precision Assembly and “Smart” Activation (DNA and Supramolecular Scaffolds)

A central advantage of nucleic acid and supramolecular scaffolds is molecular addressability, which controls the location and timing of hemin catalytic activity, reducing off-target ROS while enabling tumor-selective amplification.^27,36–39^ Compared with random conjugation, programmable architectures produce more reproducible cofactor positioning and permit logic-gated activation driven by tumor cues (enzymes, ions, metabolites), enabling “conditional nanozymes” that are better suited for in vivo use.^29,36^

G4/hemin-DNA enzymes (DNAzymes) are established heme-mimetic catalysts in which guanine-rich sequences fold into G4 structures stabilized by monovalent cations (K^+^/Na^+^), enabling hemin stacking and peroxidase-like activity.^29,30^ Activity depends strongly on the G4 topology and local sequence context; parallel folds often support more favorable hemin stacking than antiparallel forms, yielding higher turnover.^40,41^ For cancer therapy, the key value lies in coupling endogenous H_2_O_2_ to on-demand radical formation while maintaining low activity in blood/healthy tissue.^36,29^ Accordingly, modern constructs embed hemin–G4 catalysis into DNA logic gates (AND/OR/NOT; multi-input circuits) such that activation requires tumor-specific conditions.^42^ This is achieved through (i) strand-displacement/walker circuits to regulate G-rich exposure and amplify catalyst assembly;^36,43,44^ (ii) aptamer-controlled switching that couples biomarker recognition to DNAzyme formation;^45,46^ and (iii) enzyme-triggered cleavage that restricts activation to tumor enzymatic signatures.^47,48^

Supramolecular host–guest systems provide reversible assembly and microenvironment responsiveness (temperature, redox, and competitive guests), enabling stimulus-regulated therapy and controlled release.^49^ Cyclodextrins are prominent hosts that can modulate porphyrin photophysics, offering strategies to suppress off-target photosensitivity while enabling *in situ* activation.^50,51^ Representative systems highlight how precision assembly combined with tumor-specific triggers can confine hemin catalysis to malignant tissue and amplify therapeutic output (Table 1). Bi et al. engineered Hemin/DNA hairpin probes (DHPs)/Polydopamine­(PDA)@Copper sulfide­(CuS) in which elevated Apurinic/apyrimidinic endonuclease 1 (APE1) cleaves DNA hairpins to initiate catalytic hairpin assembly and generate G4–hemin–DNAzymes. This enabled tumor-selective imaging and ^•^OH-mediated CDT, which was further potentiated by a second near-infrared window (NIR-II) photothermal heating for synergistic ablation with low systemic toxicity (Figure 3).^52^ To address hypoxia, Yuan et al. designed virus-like mesoporous silica nanoparticles (MSNs) coated with a rolling circle amplification (RCA)-derived DNA nanogel containing dense G4 motifs loaded with hemin to provide CAT-like O_2_ generation, coupled with a second G4 module carrying zinc phthalocyanine (ZnPc) to drive oxygen-dependent PDT in glioma.^53^ Similarly, Xiao et al. created a carrier-free RCA “DNA flower” (CH/DF) incorporating the nucleolin-targeting DNA aptamer (AS1411) aptamer for nucleolin-targeting and colocalizing hemin and Chlorin e6 (Ce6) to integrate CAT-like hypoxia relief, O_2_-amplified PDT, and ferroptosis-promoting redox stress.^7^ Extending this logic to supramolecular chemistry, Raj et al. assembled β-CD^+^/boron-dipyrromethene (BODIPY) 2D nanosheets that recruit anionic G4/hemin–DNAzymes; following uptake and endosomal escape, hemin-derived ROS/O_2_ sustain PDT, while PDT-generated ROS further sensitize cells to hemin-driven oxidative damage, producing a self-reinforcing PDT–CDT loop.^27^ Finally, the ROS-driven nanomotor with plasmid load (RDN@PL) nanomotor demonstrated a mechanically enabled variant in which hemin decomposed tumor H_2_O_2_ to generate O_2_ gradients for active propulsion and deeper penetration, while heme oxygenase-1 (HO-1)–mediated hemin degradation triggers CO release and CRISPR/Cas9 delivery (lactate dehydrogenase A (LDHA) targeting), linking active transport with metabolic disruption and redox/gas signaling.^54^

**Figure 3:**
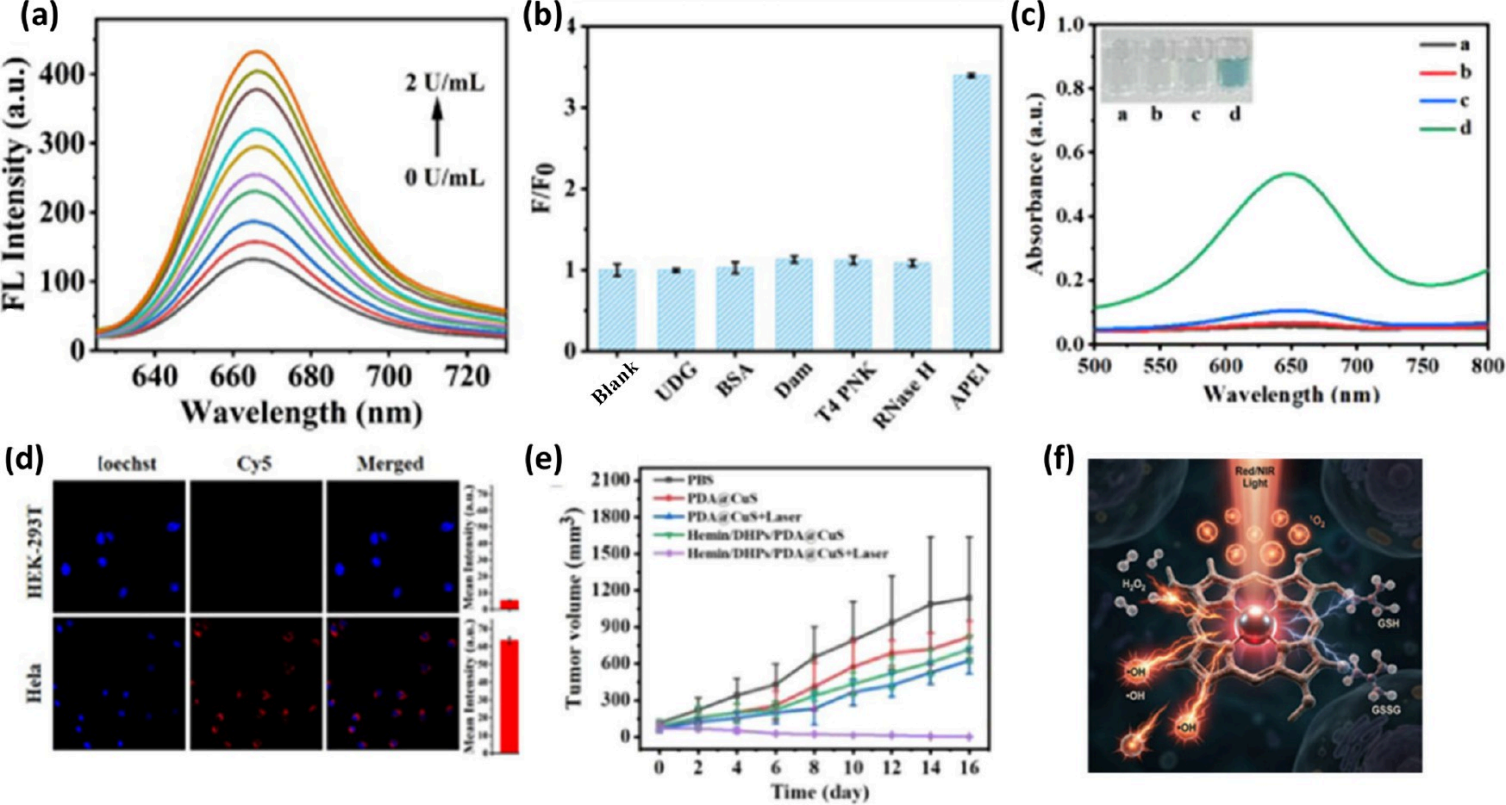
Representative precision-assembled hemin nanozyme systems enabling tumor-triggered catalytic activation. Example of an APE1-responsive DNA hairpin assembly system that generates G4–hemin DNAzymes for tumor-selective ROS production and photothermal-enhanced chemodynamic therapy. (a) Fluorescence responses of the Hemin/DHPs/PDA@CuS nanocomposite to increasing APE1 concentrations. (b) Specificity of fluorescence activation toward APE1 over other enzymes. (c) UV–vis spectra and photographs (inset) of 3,3′,5,5′-tetramethylbenzidine (TMB) oxidation under different reaction conditions, demonstrating APE1-dependent ^•^OH generation. (d) Confocal fluorescence images and corresponding quantitative analysis of APE1-activated signals in HeLa and HEK-293T cells. (e) Tumor growth curves of HeLa tumor-bearing mice following different treatments over 16 days (mean ± SD, *n* = 5). (f) Schematic illustration of APE1-activated G-quadruplex–hemin DNAzyme formation, ROS generation, and NIR-II photothermal-enhanced CDT guided by amplified fluorescence imaging. Reprinted in part with permission from ref 52. Copyright 2025, American Chemical Society.

A comparative summary of the advantages and limitations of the major hemin nanozyme scaffold classes, including DNA, polymer/MOF, and carbon frameworks, is provided in Table 2, highlighting key trade-offs in catalytic precision, stability, manufacturability, and translational feasibility.

**Table 2:**
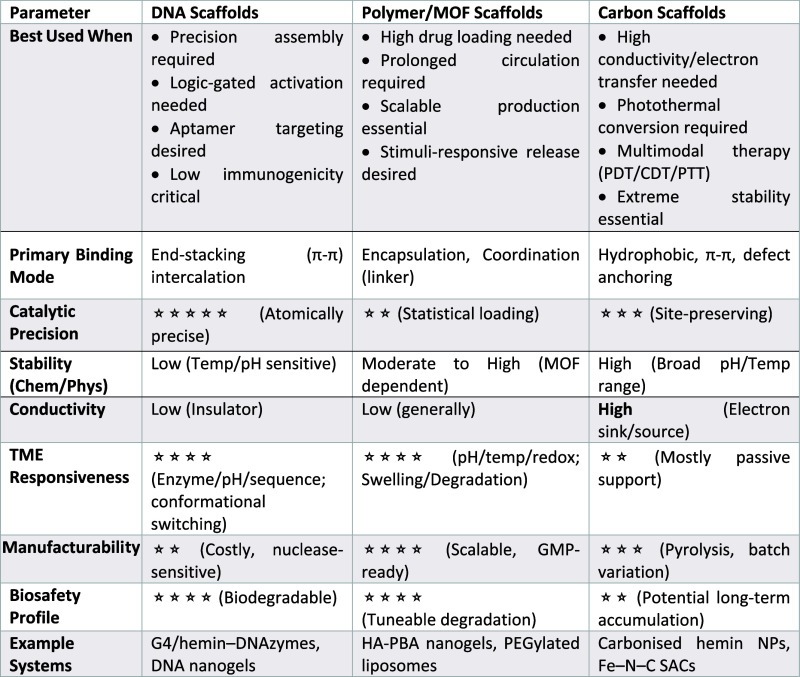
Comparative Design Matrix for Hemin-Loading Scaffolds: Structural, Catalytic, and Translational Considerations

### Material Engineering and Photophysics (Carbonized and Single-Atom Hemin Catalysts)

While scaffolds dictate the positioning of hemin, material engineering defines its properties by reshaping its electronic structure, charge-transfer kinetics, photophysics, and catalytic selectivity. Native hemin suffers from aggregation, oxidative deactivation, and inefficient charge separation, which limit its therapeutic utility.^16^ Embedding hemin within conductive or carbonized matrices stabilizes Fe–nitrogen (variable coordination) (Fe–N) active motifs, accelerates electron transport, and suppresses recombination, yielding “super-hemin” photoresponsive nanozymes capable of coupled ^•^OH and ^1^O_2_ generation. Single-atom catalysts (SACs) represent the conceptual end-point of this strategy, with isolated Fe sites (e.g., Fe–Nχ) anchored on N-doped carbon maximizes atomic utilization, enables tunable substrate activation, and reduces metal leaching.^57,58^ An overview of representative material-engineered hemin nanosystems is presented in Table 3. Lin et al. demonstrated that solvothermal carbonization converts hemin into conductive carbon architectures that markedly enhance Fenton-like kinetics, generate O_2_ to alleviate hypoxia, and support hypoxia-tolerant Type I photochemistry under irradiation, collectively amplifying ROS flux and depleting GSH for effective tumor ablation.^8,59,60^ Similarly, Li et al. reported hemin-derived carbon nanodots (HNCDs; HB nanoparticles) that preserve Fe–N_4_ coordination while shifting phototherapy toward oxygen-independent Type I pathways (O_2_^•–^/^•^OH). Beyond direct cytotoxicity, light-triggered pyroptosis and damage-associated molecular pattern release link catalytic phototherapy to systemic antitumor immunity.^28^

**Table 3:**
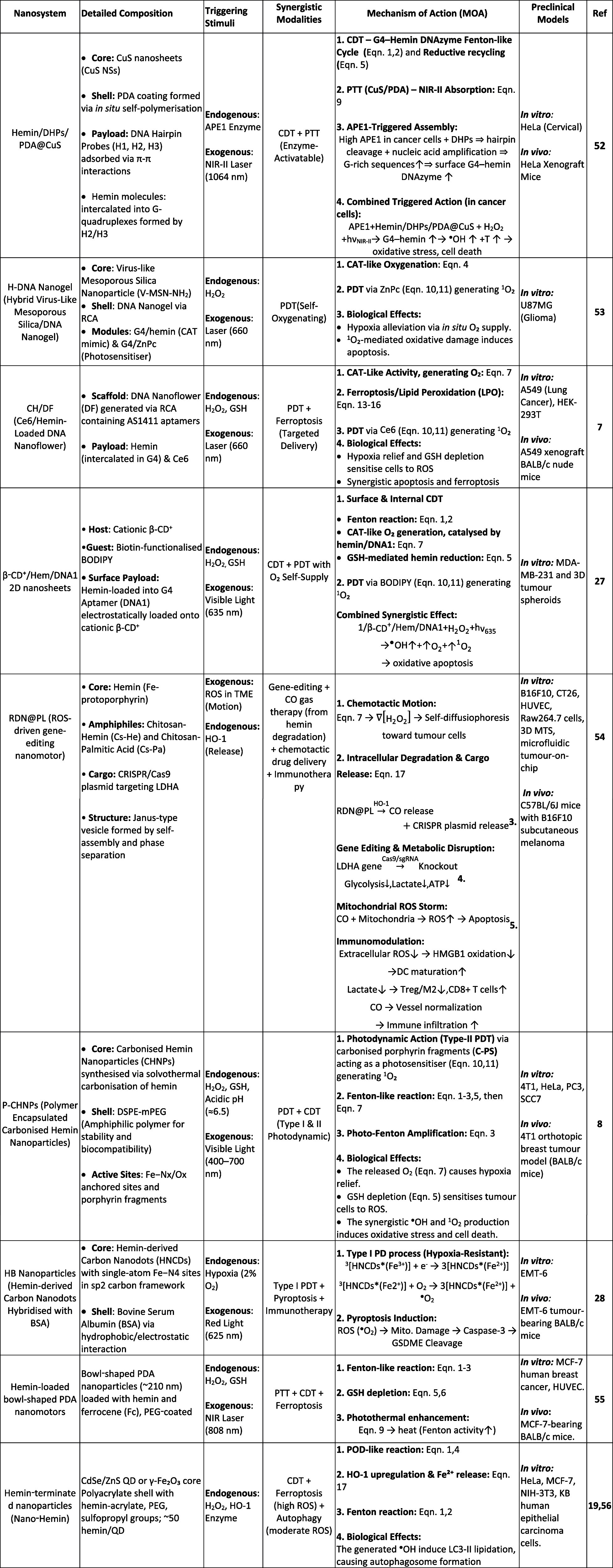
Hemin-Based Nanosystems Enabled by Precision Assembly and Material Engineering: DNA/Supramolecular Scaffolds and Carbonized or Single-Atom Hemin Platforms^a^

a Abbreviations: ^•^OH: Hydroxyl radical; ^1^O_2_: Singlet oxygen; ^3^O_2_: Triplet oxygen; 4T1: Mouse mammary carcinoma cell line; A549: Human lung adenocarcinoma cell line; APE1: Apurinic/Apyrimidinic Endonuclease 1; AS1411: Nucleolin-targeting DNA aptamer; B16F10: Mouse melanoma cell line; BODIPY: Boron-Dipyrromethene; BSA: Bovine Serum Albumin; CAT: Catalase-like activity; CDT: Chemodynamic Therapy; Ce6: Chlorin e6; CH/DF: Ce6/Hemin-loaded DNA Nanoflower; CHNPs: Carbonised Hemin Nanoparticles; Cs–He: Chitosan-Hemin; Cs–Pa: Chitosan-Palmitic Acid; CT26: Mouse colon carcinoma cell line; CuS: Copper Sulfide; DC: Dendritic Cell; DH: Deferric Hemin; DHA: Dihydroartemisinin; DHPs: DNA Hairpin Probes; EMT-6: Mouse mammary tumor cell line; FBS: Fetal Bovine Serum; G4: G-quadruplex; GOx: Glucose Oxidase; GSH/GSSG: Glutathione (reduced/oxidized); HA-PBA: Hyaluronic Acid-Phenylboronic Acid; HB: Hemin-derived Carbon Nanodots (HNCDs) with BSA; HeLa: Human cervical cancer cell line; HEK-293T: Human embryonic kidney 293 cell line; Hemin: Iron­(III) protoporphyrin IX chloride; HMGB-1: High mobility group box 1; HO-1: Heme Oxygenase-1; HUVEC: Human Umbilical Vein Endothelial Cells; ICD: Immunogenic Cell Death; KB: Human epithelial carcinoma cell line (derived from HeLa); LDHA: Lactate Dehydrogenase A; LPO: Lipid Peroxidation; MCF-7: Human breast adenocarcinoma cell line; MDA-MB-231: Human triple-negative breast cancer cell line; NIH-3T3: Mouse embryonic fibroblast cell line; NIR: Near-Infrared; O_2_^•–^: Superoxide anion; P-CHNPs: Polymer-Encapsulated Carbonised Hemin Nanoparticles; PC3: Human prostate cancer cell line; PDA: Polydopamine; PDT: Photodynamic Therapy; PEG: Polyethylene Glycol; PTT: Photothermal Therapy; QD: Quantum Dot; Raw264.7: Mouse macrophage cell line; RCA: Rolling Circle Amplification; RDN@PL: ROS-Driven Nanomotor with Plasmid Load; ROS: Reactive Oxygen Species; SCC7: Mouse squamous cell carcinoma cell line; TNBC: Triple-Negative Breast Cancer; TME: Tumor Microenvironment; Treg: Regulatory T Cell; U87MG: Human glioblastoma cell line; V-MSN: Virus-like Mesoporous Silica Nanoparticle; β-CD^+^: Cationic beta-Cyclodextrin; ZnPc: Zinc Phthalocyanine. This table summarizes representative systems discussed in the sections Precision Assembly and “Smart” Activation (DNA and Supramolecular Scaffolds) and Material Engineering and Photophysics (Carbonized and Single-Atom Hemin Catalysts).

Extending material control to active transport, Zhang et al. engineered bowl-shaped polydopamine nanomotors (HMONTs) in which cavity-driven O_2_ jet propulsion enhances tumor penetration, while near-infrared (NIR) photothermal heating accelerates hemin-mediated ROS generation; dual-Fenton designs incorporating ferrocene further amplify ^•^OH flux.^55^ At the biological interface, Sarkar et al. reframed nanohemin as an intracellular iron-programming agent by preventing dimerization and enhancing uptake; HO-1 induction liberates Fe^2+^ into the labile iron pool, enabling dose- and kinetics-dependent switching between cytoprotective autophagy and lethal ferroptosis, positioning nanohemin as a controllable iron-overload strategy.^19,56^

### Cascade Nanoreactors (Metabolic Synergism)

Cascade nanoreactors exploit tumor metabolic liabilities, elevated glucose flux, and strong dependence on antioxidant buffering, particularly GSH, to drive self-feeding therapy, in which substrate consumption (starvation) generates chemical fuel (H_2_O_2_ and protons) that amplifies hemin-catalyzed ROS production.^61,62^ GOx is frequently employed as an upstream trigger because it simultaneously perturbs metabolism, produces H_2_O_2_, and generates gluconic acid that lowers the local pH to accelerate Fenton-like reactions.^63,64^ The complete reaction network underpinning starvation–CDT coupling is summarized in Table 1. In the canonical loop, GOx converts glucose into gluconic acid and H_2_O_2_ (Table 1, No. 8), inducing energy stress, acidification, and oxidant supply, while hemin catalyzes H_2_O_2_ conversion into cytotoxic radicals (Table 1, Nos. 1–4), creating an autocatalytic destruction cycle widely used to overcome the insufficient H_2_O_2_ and suboptimal pH that constrain CDT alone.^65–67^ A central limitation is that GOx consumes O_2_, potentially aggravating hypoxia and weakening oxygen-dependent therapies; accordingly, many systems integrate oxygen-evolving or oxygen-sparing components.^68,69^

Representative designs that illustrate how these principles are implemented are shown in (Table 4). HPG@hemin-GOx replaces nucleic acids with guanosine-based tetrads assembled on a hyaluronic acid (HA)-PBA framework, mimicking G4 hemin stacking while delivering GOx to tumor cells. The system integrates glucose depletion, CAT-like O_2_ generation, POD-like ^•^OH production, and GPx-like GSH consumption, converging into ferroptosis–apoptosis hybrid cell death with tumor targeting.^70^ MIL-101­(Fe)-(doxorubicin)­DOX-dihydroartemisinin (DHA) @(Tetrakis­(4-carboxyphenyl)­porphyrin) (TCPP)/GOx@PDA (MIL-101­(Fe)-DOX-DHA@TCPP/GOx@PDA) couples GOx-driven starvation with MOF degradation, iron-mediated CDT, DHA radical amplification, DOX chemotherapy, and porphyrin-mediated PDT, forming a mutually reinforcing cascade that outperforms single-modality treatments (Figure 4).^71,72^ To address glucose-depleted tumor cores, glycogen-based nanoreactors generate intracellular glucose via α-glycosidase-mediated degradation, sustaining GOx activity and H_2_O_2_ supply for hemin-driven CDT.^39,73^ UPHGC nanoparticles integrate upconversion physics, where upconversion nanoparticles (UCNPs) convert NIR light into higher-energy emissions that activate hemin photochemistry (O_2_^•–^ generation), while GOx-induced acidification dissolves CaCO_3_ to release Ca^2+^, combining oxidative stress with calcium overload and mitochondrial collapse.^74^ HBGL liposomes further intensify ferroptosis by combining GOx-derived H_2_O_2_ with β-lapachone NQO1 cycling (Table 1, Nos. 19 and 20), while hemin drives Fenton chemistry and GSH depletion, suppressing GPx4 and inducing lipid peroxidation in 4T1 models.^75^ Finally, polyethylene glycol (PEG)-(camptothecin)­CPT/Hemin nanoparticles exploit H_2_O_2_-cleavable linkers to corelease CPT and hemin. CPT-induced ROS accelerates further release, while hemin converts the amplified H_2_O_2_ pool into ^•^OH, generating a self-amplified oxidative loop with stringent tumor activation.^76^

**Table 4:**
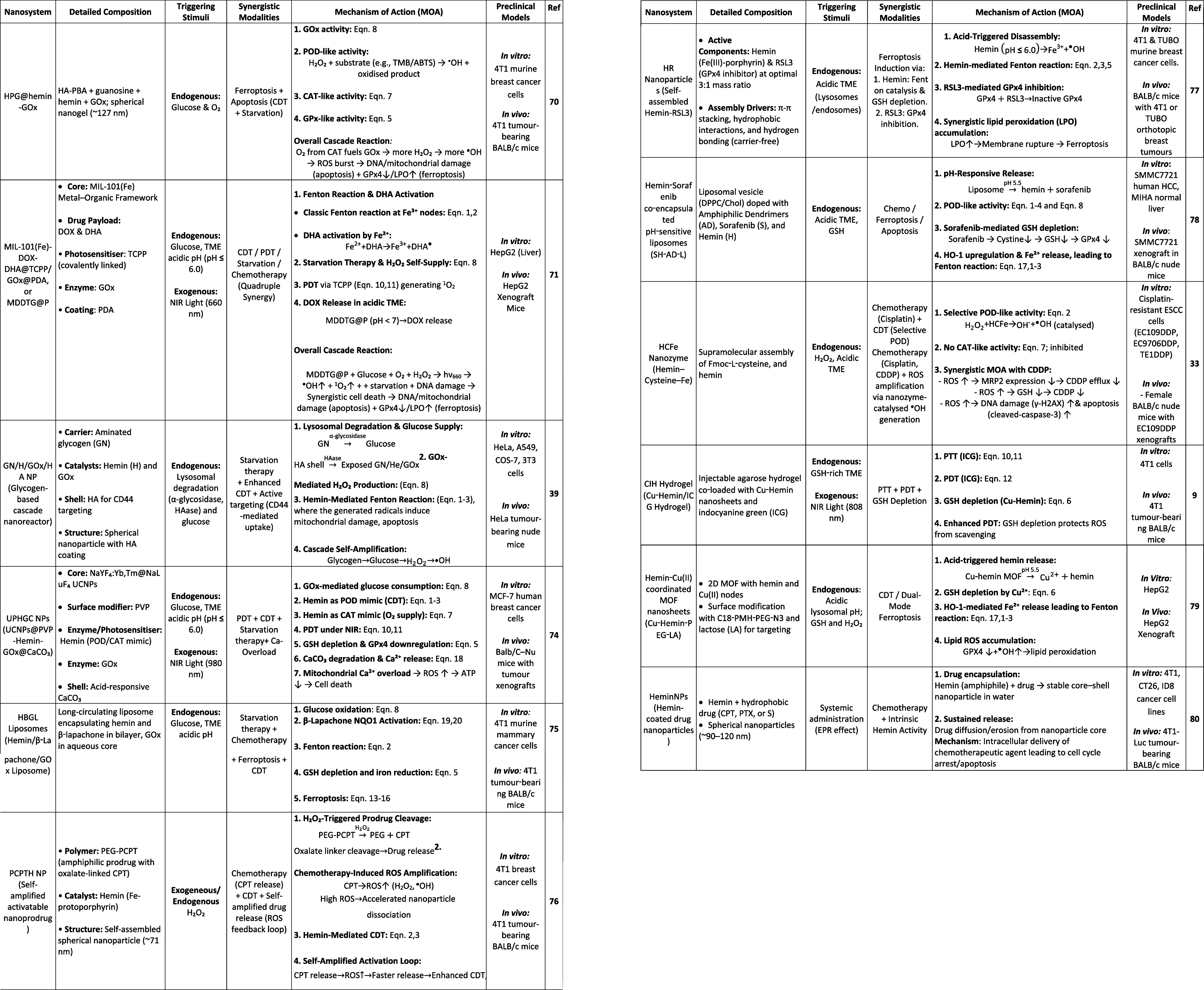
Hemin-Based Nanosystems Enabled by Cascade Nanoreactor Design and the Biological Modulation of Ferroptosis and Iron Metabolism^a^

a Abbreviations: 4T1: Mouse mammary carcinoma cell line; 4T1-Luc: 4T1 cells expressing luciferase (for bioluminescence imaging); A549: Human lung adenocarcinoma cell line; ABTS: 2,2′-Azino-bis­(3-ethylbenzothiazoline-6-sulfonic acid); AD: Amphiphilic Dendrimer (in SH-AD-L); ATP: Adenosine triphosphate; CaCO_3_: Calcium Carbonate; CAT: Catalase-like activity; CD44: Cluster of Differentiation 44 (hyaluronic acid receptor); CDDP: Cisplatin (chemotherapy drug); CDT: Chemodynamic Therapy; Chol: Cholesterol (lipid component); CIH: Cu–Hemin–ICG Hydrogel; COS-7: African green monkey kidney fibroblast cell line; CPT: Camptothecin; CT26: Mouse colon carcinoma cell line; DHA: Dihydroartemisinin; DOX: Doxorubicin; DPPC: 1,2-Dipalmitoyl-*sn*-glycero-3-phosphocholine (phospholipid); EC109: Human esophageal squamous cell carcinoma cell line; EC109DDP: Cisplatin-resistant variant of EC109 ESCC cell line; EC9706DDP: Cisplatin-resistant variant of EC9706 ESCC cell line; EPR effect: Enhanced Permeability and Retention effect; ESCC: Esophageal Squamous Cell Carcinoma; Fmoc: Fluorenylmethyloxycarbonyl; γ-H2AX: Phosphorylated histone H2AX (DNA damage marker); GN: Glycogen; GN/H/GOx/HA NP: Glycogen/Hemin/Glucose Oxidase/Hyaluronic Acid Nanoparticle; GPx: Glutathione Peroxidase-like activity; GPx4: Glutathione Peroxidase 4; GSH/GSSG: Glutathione (reduced/oxidized); HA: Hyaluronic Acid; HAase: Hyaluronidase (enzyme that degrades HA); HBGL: Hemin/β-Lapachone/GOx Liposome; HCC: Hepatocellular Carcinoma; HCFe: Hemin–Cysteine–Fe nanozyme; HeLa: Human cervical cancer cell line; HepG2: Human liver cancer cell line; HO-1: Heme Oxygenase-1; HPG@hemin-GOx: Guanosine-based polymeric nanoreactor; HR: Hemin-RSL3 self-assembled nanoparticle; ICG: Indocyanine Green; ID8: Mouse ovarian surface epithelial cell line; LA: Lactose; LPO: Lipid Peroxidation; MCF-7: Human breast adenocarcinoma cell line; MDDTG@P: MIL-101­(Fe)-DOX-DHA@TCPP/GOx@PDA (abbreviated name); MIHA: Human normal liver cell line; MIL-101­(Fe): Iron-based Metal–Organic Framework; MOF: Metal–Organic Framework; MRP2: Multidrug Resistance-Associated Protein 2 (drug efflux transporter); NIH-3T3: Mouse embryonic fibroblast cell line; NIR: Near-Infrared; NQO1: NAD­(P)­H: quinone oxidoreductase 1 (enzyme that activates β-lapachone); PBA: Phenylboronic Acid; PCPTH: PEG–PCPT/Hemin Nanoparticle (prodrug); PDA: Polydopamine; PDT: Photodynamic Therapy; PEG: Polyethylene Glycol; POD: Peroxidase-like activity; PTT: Photothermal Therapy; PTX: Paclitaxel; PVP: Polyvinylpyrrolidone; ROS: Reactive Oxygen Species; RSL3: GPx4 inhibitor; S: Sorafenib; SH-AD-L: Sorafenib-Hemin Amphiphilic Dendrimer Liposome; SMMC7721: Human hepatocellular carcinoma cell line; TMB: 3,3′,5,5′-Tetramethylbenzidine; TCPP: Tetrakis­(4-carboxyphenyl)­porphyrin; TE1: Human esophageal squamous cell carcinoma cell line; TE1DDP: Cisplatin-resistant variant of TE1 ESCC cell line; TUBO: Mouse breast cancer cell line (HER2/neu-positive); UCNPs: Upconversion Nanoparticles; UPHGC: UCNPs@PVP–Hemin–GOx@CaCO_3_. This table summarizes representative systems discussed in the sections Cascade Nanoreactors (Metabolic Synergism) and Biological Modulation (Ferroptosis and Iron Metabolism).

**Figure 4:**
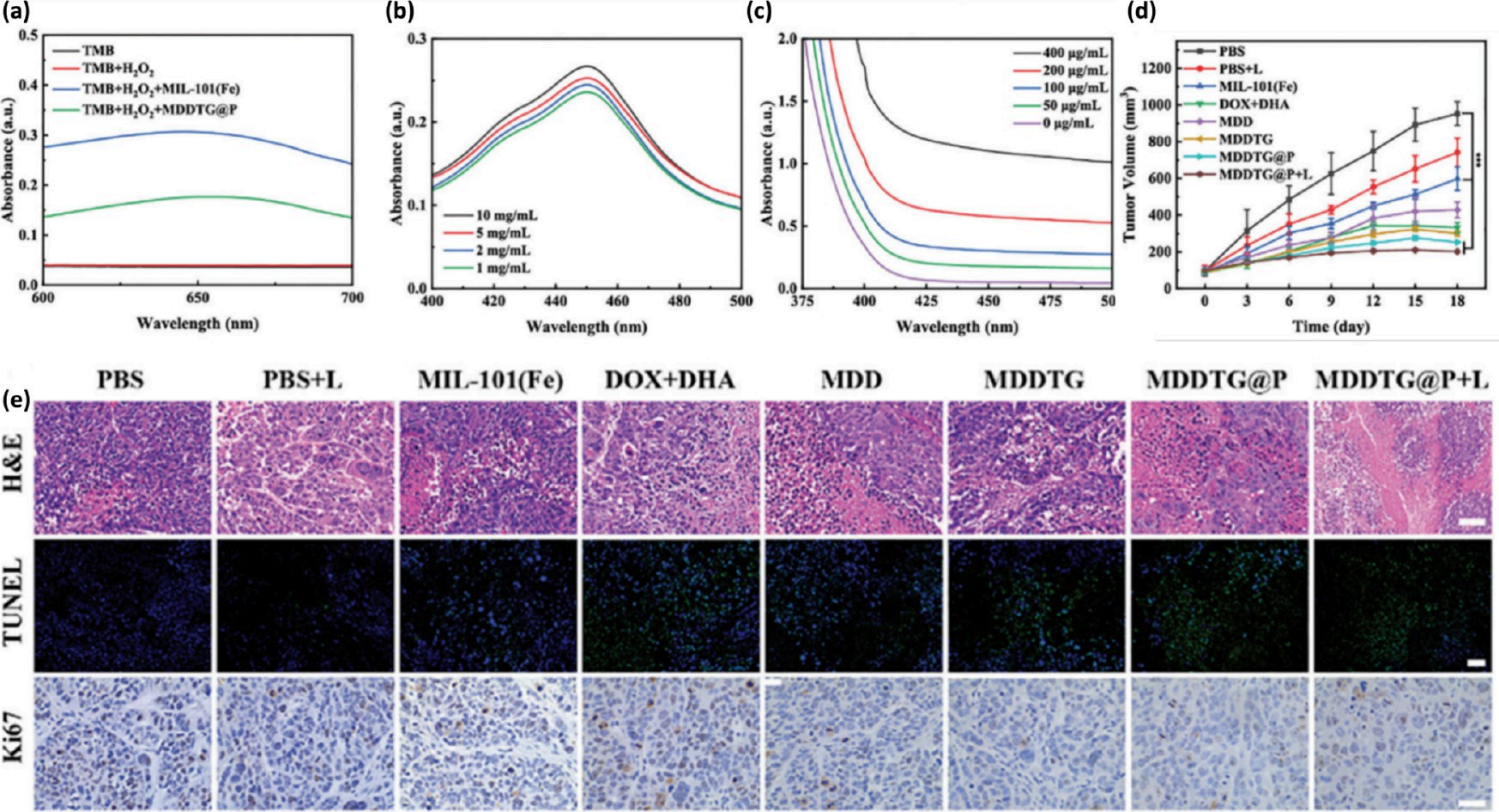
Representative cascade nanoreactor strategy for redox amplification and enhanced antitumor therapy. Example of a MIL-101­(Fe)-based nanoreactor (MDDTG@P) integrating GOx-driven metabolic amplification with Fenton-like catalysis. (A) UV–vis absorption spectra of TMB under different conditions, demonstrating Fenton-like catalytic activity in the presence of H_2_O_2_ and MIL-101­(Fe)-based nanoreactors. (B) Glucose-dependent catalytic activity of MDDTG@P, confirming GOx-mediated metabolic amplification. (C) UV–vis absorption of 5,5′-dithiobis­(2-nitrobenzoic acid) (DTNB) following GSH consumption by MDDTG@P, indicating antioxidant depletion (*n* = 3). (D) Tumor growth curves of BALB/c nude mice receiving different treatments. (E) Haematoxylin and eosin, terminal deoxynucleotidyl transferase dUTP nick end labeling, and proliferation marker Ki67 staining of tumor tissues, illustrating treatment-induced apoptosis and proliferation inhibition. Reprinted in part with permission from ref 71. Copyright 2024 John Wiley and Sons.

### Biological Modulation (Ferroptosis and Iron Metabolism)

Ferroptosis is an iron-dependent cell death program driven by the lethal accumulation of lipid peroxides.^81,82^ It is governed by cystine import through the cystine/glutamate antiporter (system Xc^–^),^83,84^ GSH biosynthesis, and GPx4-mediated lipid peroxidase detoxification.^77,85,86^ Hemin-based platforms are uniquely positioned to engage ferroptosis because they can concurrently expand the labile iron pool, catalyze radical chemistry that initiates and propagates lipid peroxidation, and weaken antioxidant defenses via GSH depletion and GPx4 suppression^19^ (reactions summarized in Table 1, Nos. 5, 6, and 13–16; HO-1 axis in No. 17).

Multiple strategies have demonstrated how ferroptosis can be rendered selective and resistance-reversing. Representative examples are summarized in Table 4. Zhou et al. reported HR, a carrier-free pH-responsive coassembly of hemin and the GPx4 inhibitor RSL3, which synergistically amplifies lipid peroxidation and ferroptosis in TNBC models with favorable biosafety.^77^ Su et al. introduced a dual-hit approach using pH-responsive liposomes coloading sorafenib (system Xc^–^ inhibition) and hemin (iron priming) to overwhelm nuclear factor erythroid 2-related factor 2/metallothionein-1G (NRF2/MT-1G) defenses and reverse sorafenib resistance in hepatocellular carcinoma.^78^ Jiang et al. addressed catalytic selectivity by engineering the POD-exclusive HCFe nanozyme (Hemin–Cysteine–Fe), suppressing CAT-like H_2_O_2_ dissipation and forcing H_2_O_2_ into ^•^OH production; the resulting ROS surge depletes GSH, downregulates efflux transporters, and restores cisplatin sensitivity in resistant ESCC.^33^ Moving toward local delivery, Zhu et al. developed injectable Cu–Hemin–ICG agarose hydrogels that combine NIR-triggered hyperthermia, reversible sol–gel release, PDT, and strong GSH scavenging by Cu–Hemin nanosheets, achieving complete tumor eradication in 4T1 models.^9^ Acid-degradable Cu-hemin-PEG-LA nanosheets further engage both classical and nonclassical ferroptosis by releasing Cu^2+^ (GSH consumption/GPx4 suppression) and hemin (HO1-driven Fe^2+^ overload), overcoming sorafenib resistance in liver cancer.^79^ Finally, hemin can function as an intrinsic amphiphilic carrier. Carrier-free HeminNPs coassemble with hydrophobic chemotherapeutics (CPT, PTX, SOR), enabling drug delivery alongside intracellular hemin release that perturbs iron and redox homeostasis, thereby sensitizing tumors, improving efficacy, and reducing systemic toxicity in 4T1 models.^80^

## Design Rules for Hemin-Based Nanoarchitectures: A Comprehensive Design Toolbox and Decision Matrix

The therapeutic case studies discussed above reveal recurring mechanistic constraints that directly inform the rational nanozyme design. By analyzing the limitations observed in cascade catalysis, scaffold stability, and tumor microenvironment heterogeneity, several practical design rules emerge that guide the engineering of next-generation hemin nanozymes.

Therapeutic studies of cascade nanoreactors combining glucose oxidase (GOx) and hemin revealed that catalytic efficiency observed in vitro often failed to translate in vivo.^87^ In these systems, GOx generates hydrogen peroxide from glucose, which hemin subsequently converts into cytotoxic hydroxyl radicals through Fenton-like reactions.^10,63^ However, when the two catalytic components are loosely codelivered, the diffusible intermediate H_2_O_2_ is rapidly scavenged by tumor antioxidant systems before reaching the secondary catalytic site.^88,89^

These observations establish a “spatial proximity design rule”: effective cascade nanozymes must enforce nanoscale confinement between sequential catalytic components. Architectures such as core–shell nanoparticles, polymer vesicles, or porous MOFs maintain sub-10–20 nm distances between enzymes, enabling rapid intermediate channelling and preventing volumetric dilution or antioxidant quenching.^90^ Such spatial confinement preserves high local concentrations of reactive intermediates and significantly enhances ROS amplification within the tumor microenvironment.

Studies of hemin nanozyme platforms also reveal that tumor microenvironment heterogeneity strongly influences scaffold performance. DNA-based nanoassemblies offer exceptional spatial precision and stimulus-responsive activation but often suffer from nuclease degradation and ionic instability during systemic circulation.^91,92^ Consequently, their therapeutic application is generally limited to localized delivery or diagnostic systems.^93^

These observations motivate a “scaffold selection design rule”: nucleic acid architectures are best suited for programmable or trigger-responsive local systems, whereas robust carbonaceous or crystalline frameworks are preferred for systemic therapies targeting heterogeneous solid tumors. Carbonized hemin materials and single-atom catalysts embedded in sp^2^-carbon matrices exhibit enhanced structural stability, sustained catalytic activity under acidic and hypoxic conditions, and improved substrate interactions, enabling persistent ROS generation within the tumor microenvironment. Together, these examples illustrate how therapeutic performance directly informs structural design principles, transforming empirical observations into a practical toolbox for engineering programmable hemin nanozymes, as will be explored in the following sections.

The maturation of hemin-based nanomedicine has driven a shift from empirical material selection toward rational, principle-driven design, in which catalytic behavior, biological response, and translational performance are deliberately programmed. Leveraging advances in nucleic acid nanotechnology, polymer/MOF engineering, carbon materials, and coordination chemistry, this section distils the literature into a design toolbox that links scaffold choice, coordination environment, and spatial organization to function. Rather than cataloguing architectures, we focus on foundational design questions: when molecular programmability justifies reduced stability; how coordination motifs (e.g., Fe–N_4_ vs Fe–N_4_S or Fe–C) redirect catalytic pathways; and how spatial and kinetic constraints govern cascade efficiency in vivo. Formalizing these considerations enables scaffold selection based on physical principles and biological objectives rather than convenience-driven material choice.

### Scaffold Selection: Structure Dictates the Function

In hemin nanoarchitectures, the scaffold is an active design element that governs thermodynamic stability, electronic coupling, and substrate accessibility. The choice between DNA, polymer/MOF, or carbon scaffolds represents the most critical decision point.

### DNA Scaffolds: Precision and Conditional Activation

DNA scaffolds, including G-quadruplex/hemin assemblies,^16,52^ RCA-derived DNA flowers,^9^ and enzyme-gated probes, offer unmatched programmability and Ångström-level spatial control over hemin positioning.^48^ These features enable logic-gated, stimulus-responsive activation, and tumor-selective catalysis. Central to these systems is the G4/hemin interface, governed by end-stacking interactions^29,41^ that preserve iron accessibility and facilitate proton transfer. Sequence-level tuning modulates redox potential and balances POD-, CAT-, and peroxygenase-like activities,^41,94,95^ a degree of control that is largely inaccessible in heterogeneous supports. DNA scaffolds are uniquely compatible with molecular logic operations,^43,45–48^ and hybrid “coronazyme” architectures further amplify the activity by combining DNA coronas with metallic cores.^96^ However, their translational utility is constrained by nuclease susceptibility and ion-dependent G4 stability,^91,92^ necessitating protective strategies or hybridization. Thus, DNA scaffolds trade maximum precision for reduced stability and scalability issues.

### Polymer and MOF Scaffolds: Confinement and Cascade Efficiency

Polymer and MOF scaffolds occupy a middle ground between DNA precision and carbon robustness, respectively. Polymeric systems, such as HA-based assemblies,^39^ PBA-modified networks,^97^ dendrimers,^78^ hydrogels,^98^ and carrier-free hemin amphiphiles,^99^ excel in biocompatibility, tumor targeting, and modular codelivery, making them well-suited for cascade nanoreactors requiring sustained enzyme–hemin proximity. Their limitations lie in reduced structural precision and diffusion-limited catalysis in dense matrices.

MOFs provide crystalline confinement with a high active site density when hemin or porphyrinic ligands are used as structural components.^100,101^ Size-selective pores admit small substrates while excluding deactivating biomacromolecules,^102^ and acid-stable frameworks retain activity in tumor or lysosomal environments.^101^ These attributes favor MOFs for multimodal nanoreactors that integrate CDT, starvation therapy, and drug activation.

### Carbon Scaffolds: Electronic Coupling and Robust Catalysis

Carbon-based platforms, including carbonized hemin, graphene derivatives, carbon dots, carbon nanotubes (CNTs), and single-atom Fe–N_4_ motifs, prioritize electron transfer efficiency, durability, and hypoxia tolerance.^103,104^ Strong π–π interactions suppress hemin aggregation and accelerate Fe^3+^/Fe^2+^ cycling,^18,107,108^ enabling persistent radical generation and Type I photochemistry under oxygen-limited conditions.^105,106^ Carbon scaffolds also act as substrate concentrators and redox mediators. Graphene oxide (GO) functional groups can facilitate H_2_O_2_ decomposition,^109^ while carbon dots promote rapid charge-transfer and stabilize reactive intermediates.^8,59,110^ Their exceptional environmental robustness supports long-lived catalysis under harsh biological conditions.^109,111^ However, their limited biodegradability and potential accumulation necessitate careful surface engineering to balance efficacy and biosafety.^112–114^

### The Electronic Engine: Coordination Chemistry as a Selectivity Dial

Once the scaffold is defined, the local coordination environment of the iron center becomes the dominant determinant of catalytic selectivity. In hemin-based nanozymes, the balance between POD, CAT, OXD, and superoxide dismutase (SOD) activities is not stochastic but is dictated by the ligand field, axial coordination, and electronic coupling at the Fe site.

### Fe–N_4_ as Baseline Coordination Motif

The planar Fe–N_4_ porphyrinic motif defines the electronic baseline of the hemin nanoarchitectures. Ligand field splitting places the dz^2^ orbital perpendicular to the porphyrin plane as the primary substrate-binding axis, enabling the activation of H_2_O_2_ via heterolytic O–O cleavage to form high-valent oxo–ferryl intermediates (Compound I), which underpin POD activity.^115,116^ In the absence of axial modulation, Fe–N_4_ typically exhibits mixed POD/CAT behavior, with pathway dominance governed by pH, substrate concentration, and steric confinement.^117,118^ Spatial restriction within G4 DNA pockets or composite matrices biases Fe–N_4_ toward enhanced POD kinetics by increasing substrate enrichment and limiting axial ligand access.^118^ Thus, Fe–N_4_ provides a robust and versatile platform for balanced ROS generation and oxygen management, particularly in CDT–PDT hybrid systems.

### Axial Modulation as a Catalytic Dial

Beyond scaffold effects, axial ligand engineering enables deterministic control over the redox potential, spin state, and reaction selectivity. Across the reported systems, Fe–N_4_, Fe–N_4_S, and Fe–C/Fe–N–C motifs function as practical catalytic dials.

### Fe–N_4_ and Fe–N_4_Cl (Heme-like Coordination)

Native Fe–N_4_ centers support tunable POD/CAT activity depending on confinement and the microenvironment.^119,120^ The introduction of an axial chloride ligand (Fe–N_4_Cl) lowers the dz^2^ orbital energy and reduces the activation barrier for ^•^OH generation, markedly enhancing POD kinetics.^31,32^ This kinetic bias drives intense oxidative stress and lipid peroxidation, rendering Fe–N_4_Cl single-atom nanozymes particularly effective for ferroptosis induction and tumor eradication.^31^

### Fe–N_4_S (Axial Sulfur)

Axial sulfur coordination, typically via cysteine thiols, imposes a strong-field covalent ligand environment reminiscent of cytochrome P450 systems.^121^ This coordination blocks the second H_2_O_2_ binding step required for CAT activity, enforcing selectivity-locked POD behavior and quantitative channelling of H_2_O_2_ into ^•^OH production.^33^ Fe–N_4_S architectures are therefore ideally suited for chemoresistance reversal and therapies that require sustained high ROS flux.^23,122^

### Fe–N_5_/Fe–N_6_ (Axial Nitrogen Ligation)

Axial nitrogen donors (imidazole, pyridine, and graphitic N) stabilize high-valent iron intermediates and suppress uncontrolled radical leakage.^123,124^ These motifs favor OXD-like activity, oxygen consumption, and long-term catalytic durability, making them better suited for oxygen modulation and stability-driven nanozymes rather than aggressive ROS amplification.

### Carbon-Embedded Iron: Hypoxia-Resilient Electron Transfer

Carbon-embedded Fe–C and Fe–N–C motifs, including carbonized hemin and SAC, couple the iron center to an sp^2^ carbon lattice, enhancing charge delocalization and electron transfer kinetics.^7,125–127^ These architectures preferentially promote Type I photochemistry, generating O_2_^•–^ and ^•^OH via electron transfer pathways that remain effective under severe hypoxia.^128,129^ Conductive carbon matrices also suppress hemin aggregation and oxidative deactivation, enabling photoenhanced CDT with superior robustness compared to other nanocarriers.

### Defect Engineering and Coordination Vacancies

Further selectivity control arises from defect engineering. Fe–N_3_ sites, characterized by coordination vacancies, create electron-deficient centers that preferentially catalyze SOD- and CAT-like reactions, enabling ROS scavenging without cytotoxic intermediates.^130^ These motifs are better suited for cytoprotective and anti-inflammatory applications than the other two. In contrast, metallic Fe–C motifs (e.g., Fe_3_C and Fe–C single atoms) exhibit broad OXD/POD activity with reduced selectivity, favoring sensing, antibacterial, or nonselective oxidative applications.^128,129,131^

### Design Rule Summary

Collectively, these coordination environments function as programmable catalytic dials. Fe–N_4_ provides a versatile baseline. Axial Cl^–^ or S ligation biases the activity toward aggressive oxidative catalysis for ferroptosis or chemoresistance reversal. Axial N ligation favors stability and oxygenation modulation. Carbon-embedded Fe–C/Fe–N–C motifs unlock hypoxia-resilient, photoenhanced electron-transfer pathways. Therefore, rational tuning of the iron coordination sphere enables hemin nanoarchitectures to be precisely matched to therapeutic objectives by controlling the intensity, selectivity, and durability of redox activity (Table 5).

**Table 5:** Coordination Engineering of Hemin Nanozymes: Activity Programming and Therapeutic Targeting

Coordination Motif	Dominant Activity	Selectivity Mechanism	Therapeutic Context	Design Rule/Application
Fe–N_4_(Planar Heme)	POD/CAT (1:1)	Mixed H_2_O_2_ activation: Partial O–O bond cleavage vs full dissociation	Baseline reference, Dual ROS/O_2_ supply	Use for balanced therapy when both ROS generation and hypoxia relief are needed
Fe–N_4_Cl (Axial Cl)	POD Enhanced	Axial Cl^–^ ligand modulates d-band center, lowers ^•^OH generation barrier (ΔE↓)	Maximal ROS generation, Ferroptosis induction	Select for aggressive tumor therapy when maximal oxidative damage is desired
Fe–N_4_S (Axial S)	POD-Exclusive	Sulfur coordination (1) locks Fe in high-spin-state, (2) suppresses CAT pathway by blocking O_2_ release channel	Drug-resistant tumors, Hypoxic TME (prevents wasted O_2_ generation)	Use when CAT activity is counterproductive; ideal for chemo-sensitization
Fe–N_3_(Vacancy)	SOD/CAT Enhanced	Lower coordination barrier for ROS decomposition; increased Fe accessibility	Cytoprotection, Anti-inflammatory therapy	Employ in normal tissue protection or noncancer applications (psoriasis, wound healing)
Fe–N_5_(Axial N)	OXD/Stability	Axial N stabilizes Fe(IV)=O intermediates, promotes O_2_ binding without release	Oxygen consumption, Hypoxia induction	Use for metabolic competition (starving aerobic processes) or stable catalysis
Fe–C (Carbon-Embedded)	Type I Photodynamic + PTT	Carbon matrix enables e^–^ transfer, suppresses charge recombination	Deep-tissue penetration, Photothermal synergy	Select for multimodal therapy (PDT/CDT/PTT) with enhanced tissue penetration

### Spatiotemporal Design Rules for Cascade Nanoreactors

Biological cascades rely on substrate channeling within metabolons to overcome diffusion limits and protect reactive intermediates. Replicating this behavior in hemin-based nanoreactors (e.g., GOx-hemin cascades) requires strict spatiotemporal control over the enzyme positioning, confinement, and reaction kinetics.

### Spatial Colocalization and Proximity Channelling

The cascade efficiency is governed by nanoscale proximity and intermediate retention. For diffusible intermediates such as H_2_O_2_ generated by GOx (Table 1, No. 8), catalytic enhancement occurs only when production and consumption occur within a confined regime. DNA origami studies have shown that positioning GOx and downstream peroxidases or hemin mimics at ∼10 nm separation yields up to ∼15-fold cascade enhancement by enabling proximity channelling, whereas separations >20 nm collapse efficiency due to volumetric dilution (∝*r* ^3^) and scavenging.^90^ Local physicochemical effects further contribute to this phenomenon; the negative charge of DNA lowers the local pH, enhancing GOx and POD activity.^132^ Accordingly, high-performance cascade nanoreactors must enforce <20 nm enzyme–catalyst spacing and controlled microenvironments, favoring coencapsulation strategies (MOFs, liposomes, polymer nanoreactors) over random co-immobilization. Compartmental localization further dictates the outcome. Lysosomal positioning promotes acid-triggered iron release and Fenton activation,^133^ cytosolic localization sustains redox cycling and GSH depletion,^134^ and mitochondrial proximity maximizes oxidative collapse.^135^ At the tissue scale, substrate availability must be anticipated; in glucose- or oxygen-poor tumors, self-fuelling strategies (e.g., glycogen-derived glucose reservoirs or dual H_2_O_2_ sources) are required to maintain cascade continuity. Collectively, cascade systems must function as closed microreactors, in which H_2_O_2_ generation, conversion, and antioxidant depletion are spatially colocalized and metabolically sustained.

### Confinement, Diffusion, and Kinetic Matching

When atomic-scale positioning is impractical, confinement-based designs can regulate diffusion and reaction coupling. Encapsulation within porous shells (e.g., hollow silica or MOFs) increases the collision frequency while preserving local H_2_O_2_ gradients. A key rule is pore size matching: pores must admit primary substrates (glucose, O_2_) while retarding the escape of intermediates.^136,137^ Reaction–diffusion modeling has revealed a core–shell paradox: overly active cores can flood confined volumes with intermediates, suppressing net turnover, whereas architectures pairing a moderate generator core with a catalyst-rich shell achieve higher flux by balancing production and consumption rates.^138^ Thus, effective confinement requires tuning of both the spatial arrangement and the relative catalytic activities. Design priorities diverge according to the application. In biosensing, approximately 10 nm colocalization maximizes signal transduction and lowers the detection limits. In cancer therapy, confinement within MOFs or polymer micelles protects enzymes during circulation while enforcing *in situ* H_2_O_2_ conversion to ^•^OH at tumors, minimizing systemic oxidative toxicity.^139^

### Engineering Trade-Offs: Activity, Safety, and Scale

High catalytic activity inevitably raises concerns regarding biosafety. The activity–biosafety paradox arises because POD-driven ROS required for tumor eradication can damage healthy tissues. A common solution exploits physiological pH gradients: nanozymes are engineered to remain inactive or CAT-like at pH 7.4 but switch to POD-dominant toxicity in acidic tumors (pH ≈ 6.5).^139^ Hemin leaching represents another failure mode; robust designs require strong anchoring or confinement, whereas protein corona engineering (e.g., albumin-coated GO) can sequester leaked hemin and buffer inflammation.^140,141^

Economic constraints impose a trade-off between scalability and precision. Carbon-supported and single-atom nanozymes offer high atom utilization and scalable synthesis, favoring bulk or high-dose applications,^142,143^ albeit with reduced site uniformity. In contrast, DNA-based nanozymes provide unmatched programmability and biodegradability at a higher material cost.^144^ For high-value applications, this cost is acceptable: assembled hemin–DNAzymes can achieve catalytic costs below 10% of natural horseradish POD owing to their superior stability and reusability.^145^

### Design Rule Summary

Hemin-based cascade nanoreactors are governed by the physical laws of proximity, confinement, and kinetics rather than empirical assembly. Optimal systems enforce nanoscale colocalization, balanced reaction–diffusion coupling, and context-dependent safety gating. As summarized in Table 6 and Figure 5, these rules provide a roadmap for the rational construction of next-generation hemin nanozymes, enabling predictable performance in diagnostics, therapy, and catalysis.

**Figure 5:**
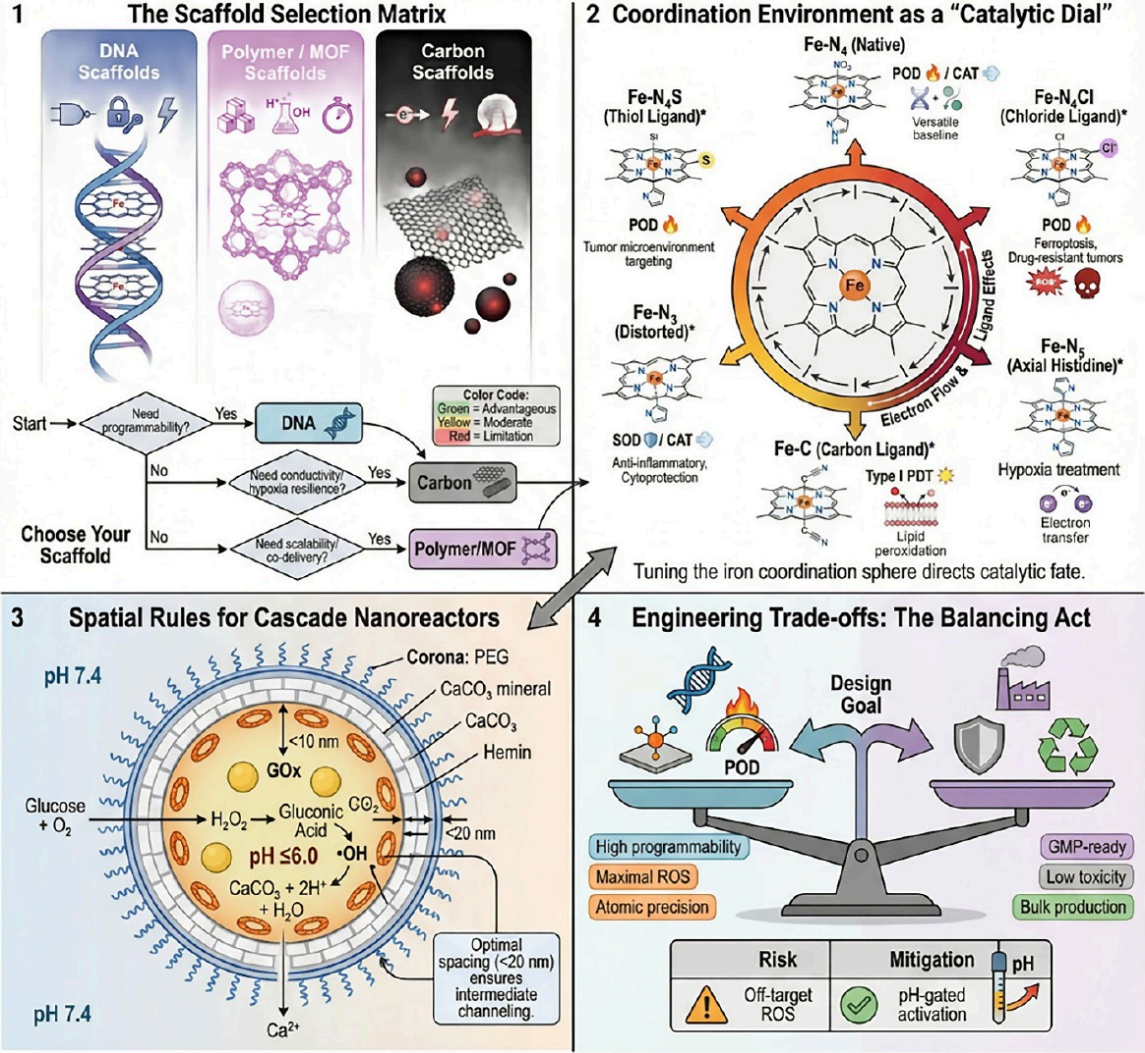
Design toolbox for hemin-based nanozymes: (1) scaffold selection matrix, (2) coordination environment as a catalytic dial, (3) spatial design rules for cascade nanoreactors, and (4) translational engineering trade-offs. *Created by illustrae.co*.

**Table 6:** Decision Matrix for Programming Hemin Nanozyme Activity, Selectivity, and Translational Performance

Design Parameter	Rule/Heuristic	Mechanism/Rationale	Ideal Application
Scaffold Choice	G-Quadruplex DNA	Precise end-stacking; tunable p*K*_a_; Programmable	Diagnostics: Logic Gates; Precision Sensing
	GO	2D surface π-π-stacking; substrate concentration	Biointerfaces: Cell scaffolds; Surface catalysis
	MOF/Polymer	Confinement; pH-responsive switching	Smart drug delivery: Stimuli-responsive therapy.
Active Site	Fe–N_4_Cl (Axial Cl)	Lowers ^•^OH generation barrier (Electronic)	Maximal ROS: Ferroptosis (Cancer)
	Fe–N_4_S (Axial S)	Spin-state lock; suppresses CAT activity	Drug-Resistant Tumors: Hypoxic therapy
	Fe–N_3_(Vacancy)	Low coordination; facilitates ROS scavenging	Cytoprotection: Anti-inflammatory (Psoriasis)
Cascade Design	Distance <20 nm	Proximity channelling of intermediates (H_2_O_2_)	High-efficiency biosensors
	Core–Shell	Confined reactor; prevents intermediate loss	In vivo nanoreactors
Biosafety	pH-Gating	Inactive at pH 7.4; Active at pH 5.5	Systemic administration
	Albumin Corona	Sequesters free hemin; prevents hemolysis	Blood-contacting materials
Scalability	Pyrolyzed Fe–N–C	Low-cost precursors; stable active sites	Environmental/Bulk therapy
	Synthesized DNA	High precision; cost-effective	*In vitro* diagnostics

Hemin-based nanoarchitectures have evolved from simple porphyrin carriers into highly programmable catalytic systems capable of overcoming the multifactorial barriers of the TME. As highlighted throughout this Review, the unique redox-active nature of hemin enables the simultaneous modulation of ROS, oxygen availability, GSH buffering, and iron metabolism, which are key vulnerabilities that collectively limit the efficacy of conventional cancer therapies. By integrating chemodynamic, photodynamic, ferroptotic, metabolic, and immunomodulatory mechanisms within a single nanoscale construct, hemin nanozymes exemplify how catalytic nanomedicine can move beyond single-modality interventions toward system-level therapeutic control.

A central theme emerging from recent advances is that therapeutic performance is no longer dictated by hemin alone but by how its activity is programmed through nanoscale engineering. Precision DNA and supramolecular scaffolds offer unparalleled control over catalytic activation and logic gating, enabling tumor-selective and stimulus-responsive redox amplification. Polymer and MOF platforms provide confinement, pharmacokinetic tunability, and cascade compatibility, bridging molecular precision and translational feasibility. Carbon-based and single-atom architectures fundamentally reshape hemin photophysics and electron transfer pathways, unlocking hypoxia-resistant Type I photochemistry and long-lived catalytic robustness. Across these platforms, coordination chemistry, particularly axial ligand and defect engineering, has emerged as a powerful “catalytic dial”, enabling deliberate selection between antioxidant, oxygen-evolving, or aggressively pro-oxidant reaction pathways.

This Review consolidates a set of unifying spatiotemporal and translational design rules. Efficient cascade nanoreactors require nanometer-scale colocalization of catalytic components to retain diffusible intermediates, whereas confinement strategies must balance production and consumption kinetics to avoid reaction flooding. At the biological interface, safety is governed not by catalytic suppression but by conditional activation through pH-gating, compartmentalization, and protein-corona-mediated buffering, allowing high intratumoral activity without systemic toxicity. Finally, scalability and economic considerations delineate distinct application niches, positioning DNA-based systems for high-value precision diagnostics and carbon-based or polymeric nanozymes for robust and large-scale therapeutic deployment.

Collectively, these insights mark a transition of hemin nanomedicine from empirical material selection to a predictive, rule-based engineering discipline. Rather than asking whether hemin should be used, future designs must focus on how its coordination environment, scaffold context, and spatial organization are orchestrated to match specific biological objectives. By formalizing these principles into a practical design toolbox and decision matrix, this study provides a roadmap for the rational development of next-generation hemin nanozymes and catalytic systems capable of overcoming tumor heterogeneity, therapeutic resistance, and translational bottlenecks through physics-guided nanoengineering.

These multifactorial barriers underscore the need for catalytic nanomaterials capable of simultaneously modulating redox balance, oxygen availability, and iron metabolism within the tumor microenvironment, requirements that have positioned hemin-based nanoarchitectures as particularly promising therapeutic candidates.

## References

[1] Une N., Takano-Kasuya M., Kitamura N., Ohta M., Inose T., Kato C., Nishimura R., Tada H., Miyagi S., Ishida T., Unno M., Kamei T., Gonda K. (2021). The Anti-Angiogenic
Agent Lenvatinib Induces Tumor Vessel Normalization and Enhances Radiosensitivity
in Hepatocellular Tumors. Med. Oncol.

[2] Anemone A., Consolino L., Conti L., Irrera P., Hsu M. Y., Villano D., Dastrù W., Porporato P. E., Cavallo F., Longo D. L. (2021). Tumour
Acidosis Evaluated in Vivo
by MRI-CEST pH Imaging Reveals Breast Cancer Metastatic Potential. Br. J. Cancer.

[3] Liu Y., Zhen W., Jin L., Zhang S., Sun G., Zhang T., Xu X., Song S., Wang Y., Liu J., Zhang H. (2018). All-in-One
Theranostic Nanoagent with Enhanced Reactive
Oxygen Species Generation and Modulating Tumor Microenvironment Ability
for Effective Tumor Eradication. ACS Nano.

[4] He Y., Li Z., Cong C., Ye F., Yang J., Zhang X., Yuan Y., Ma Z., Zhang K., Lin Y., Zheng L., Liang X.-J., Gao D. (2021). Pyroelectric Catalysis-Based
“Nano-Lymphatic” Reduces Tumor Interstitial Pressure
for Enhanced Penetration and Hydrodynamic Therapy. ACS Nano.

[5] Zhou Z., Liang H., Yang R., Yang Y., Dong J., Di Y., Sun M. (2022). Glutathione
Depletion-Induced Activation of Dimersomes
for Potentiating the Ferroptosis and Immunotherapy of “Cold”
Tumor. Angew. Chem..

[6] Xiong Y., Xiao C., Li Z., Yang X. (2021). Engineering Nanomedicine
for Glutathione Depletion-Augmented Cancer Therapy. Chem. Soc. Rev..

[7] Xiao X., Chen M., Zhang Y., Li L., Peng Y., Li J., Zhou W. (2022). Hemin-Incorporating
DNA Nanozyme Enabling Catalytic
Oxygenation and GSH Depletion for Enhanced Photodynamic Therapy and
Synergistic Tumor Ferroptosis. J. Nanobiotechnol.

[8] Lin L., Pang W., Jiang X., Ding S., Wei X., Gu B. (2022). Light Amplified Oxidative
Stress in Tumor Microenvironment by Carbonized
Hemin Nanoparticles for Boosting Photodynamic Anticancer Therapy. Light Sci. Appl..

[9] Zhu S., Wang S., Liu C., Lyu M., Huang Q. (2022). Cu-Hemin Nanosheets
and Indocyanine Green Co-Loaded Hydrogel for Photothermal Therapy
and Amplified Photodynamic Therapy. Front. Oncol..

[10] Xue X., Lindstrom A., Li Y. (2019). Porphyrin-Based Nanomedicines for
Cancer Treatment. Bioconjugate Chem..

[11] Yang L., Liu Y., Ren X., Jia R., Si L., Bao J., Shi Y., Sun J., Zhong Y., Duan P.-C., Yang X., Zhu R., Jia Y., Bai F. (2024). Microemulsion-Assisted Self-Assembly
of Indium Porphyrin Photosensitizers with Enhanced Photodynamic Therapy. ACS Nano.

[12] Liang X., Chen M., Bhattarai P., Hameed S., Dai Z. (2020). Perfluorocarbon@Porphyrin
Nanoparticles for Tumor Hypoxia Relief to Enhance Photodynamic Therapy
against Liver Metastasis of Colon Cancer. ACS
Nano.

[13] Wang H., Yu D., Fang J., Cao C., Liu Z., Ren J., Qu X. (2019). Renal-Clearable Porphyrinic Metal–Organic Framework Nanodots
for Enhanced Photodynamic Therapy. ACS Nano.

[14] Li Y.-X., Liu Y., Wang H., Li Z.-T., Zhang D.-W. (2022). Water-Soluble Porphyrin-Based
Nanoparticles Derived from Electrostatic Interaction for Enhanced
Photodynamic Therapy. ACS Appl. Bio Mater..

[15] Guo J., Liu Y., Zha J., Han H., Chen Y., Jia Z. (2021). Enhancing
the Peroxidase-Mimicking Activity of Hemin by Covalent Immobilization
in Polymer Nanogels. Polym. Chem..

[16] Alsharabasy A. M., Pandit A., Farràs P. (2021). Recent Advances
in the Design and
Sensing Applications of Hemin/Coordination Polymer-Based Nanocomposites. Adv. Mater..

[17] Yang J., Xiong L., Li M., Xiao J., Geng X., Wang B., Sun Q. (2019). Preparation and Characterization
of Tadpole- and Sphere-Shaped Hemin Nanoparticles for Enhanced Solubility. Nanoscale Res. Lett..

[18] Alsharabasy A. M., Cherukaraveedu D., Warneke J., Warneke Z., Galán-Mascarós J. R., Glynn S. A., Farràs P., Pandit A. (2024). Facile Synthesis of
Hemin Derivatives with Modulated Aggregation Behaviour and Enhanced
Nitric-Oxide Scavenging Properties as New Therapeutics for Breast
Cancer. Small Sci..

[19] Sarkar A. R., Mukherjee N., Sarkar A. K., Jana N. R. (2024). Designing Nano-Hemin
for Ferroptosis-Mediated Cell Death via Enzymatic Hemin Digestion. ACS Appl. Mater. Interfaces.

[20] Fan C., Yuan J., Guo J., Kang X. (2022). Soy Protein Isolate
(SPI)-Hemin Complex Nanoparticles as a Novel Water-Soluble Iron-Fortifier:
Fabrication, Formation Mechanism and in Vitro Bioavailability. Food Bioscience.

[21] Blagodarov S. V., Zheltukhina G. A., Romanova Y. M., Alekseeva N. V., Iskhakova L. D., Semashko M. I., Tolordava E. R., Nebolsin V. E. (2022). Improving the Synthesis of Hemin Derivatives and Their
Effect on Bacterial Biofilms. J. Porphyr. Phthalocyanines.

[22] Li Z., Deng X., Hong X., Zhao S. (2022). Nanozyme Based on Dispersion
of Hemin by Graphene Quantum Dots for Colorimetric Detection of Glutathione. Molecules.

[23] Sun R., Liu R., Tian Y., Li Y., Fan B., Li S. (2025). Removing Barriers
to Tumor ‘Oxygenation’: Depleting Glutathione Nanozymes
in Cancer Therapy. Int. J. Nanomedicine.

[24] Alsharabasy A. M., Lagarias P. I., Papavasileiou K. D., Afantitis A., Farràs P., Glynn S., Pandit A. (2024). Examining
Hemin and
Its Derivatives: Induction of Heme-Oxygenase-1 Activity and Oxidative
Stress in Breast Cancer Cells through Collaborative Experimental Analysis
and Molecular Dynamics Simulations. J. Med.
Chem..

[25] Lin L., Pang W., Jiang X., Ding S., Wei X., Gu B. (2022). Light Amplified Oxidative Stress in Tumor Microenvironment by Carbonized
Hemin Nanoparticles for Boosting Photodynamic Anticancer Therapy. Light Sci. Appl..

[26] Liang C., Zhang X., Yang M., Wang W., Chen P., Dong X. (2020). Remodeling Tumor Microenvironment
by Multifunctional Nanoassemblies
for Enhanced Photodynamic Cancer Therapy. ACS
Materials Lett..

[27] Raj G., Ghosh T., D. S. V., P. H., Kumar D. B., Prasad J., V. B. A., S. M. A., Varghese R. (2024). G4-Hemin-Loaded
2D Nanosheets for Combined and Targeted Chemo-Photodynamic Cancer
Therapy. Nanoscale.

[28] Li W., Li H., Jiang G., Yang L., Nie H., Lin C., Gao W., Huang R. (2025). Creating Single Atomic Coordination for Hypoxia-Resistant
Pyroptosis Nano-Inducer to Boost Anti-Tumor Immunotherapy. Adv. Mater..

[29] Liu Z.-L., Tao C.-A., Wang J.-F. (2020). Progress on Applications of G-Quadruplex
in Biochemical Analysis. Chinese Journal of
Analytical Chemistry.

[30] Xu J., Jiang R., He H., Ma C., Tang Z. (2021). Recent Advances
on G-Quadruplex for Biosensing, Bioimaging and Cancer Therapy. TrAC Trends in Analytical Chemistry.

[31] Yin, M. ; Wang, B.-H. ; Wang, H. ; Ouyang, J. ; Hu, X. ; Wang, X. ; Liu, Y. ; Xu, F. ; Chen, Y. ; Yin, S.-F. Chlorine-Coordinated Iron Single-Atom Nanozymes for Amplified Ferroptosis in Triple-Negative Breast Cancer Therapy. In Review October 6, 2025. 10.21203/rs.3.rs-7165005/v1. PMC13041488 41736040

[32] Wei S., Sun M., Huang J., Chen Z., Wang X., Gao L., Zhang J. (2024). Axial Chlorination Engineering of Single-Atom Nanozyme: Fe-N4 Cl
Catalytic Sites for Efficient Peroxidase-Mimicking. J. Am. Chem. Soc..

[33] Zhang S., Gao X. J., Ma Y., Song K., Ge M., Ma S., Zhang L., Yuan Y., Jiang W., Wu Z., Gao L., Yan X., Jiang B. (2024). A Bioinspired Sulfur–Fe–Heme
Nanozyme with Selective Peroxidase-like Activity for Enhanced Tumor
Chemotherapy. Nat. Commun..

[34] Xu Y., Xue D., Bankhead A. I., Neamati N. (2020). Why All the Fuss about Oxidative
Phosphorylation (OXPHOS)?. J. Med. Chem..

[35] Wu F., Liu Y., Cheng H., Meng Y., Shi J., Chen Y., Wu Y. (2021). Enhanced Cancer Starvation Therapy Based on Glucose Oxidase/3-Methyladenine-Loaded
Dendritic Mesoporous OrganoSilicon Nanoparticles. Biomolecules.

[36] Sun P., Gou H., Che X., Chen G., Feng C. (2024). Recent Advances in
DNAzymes for Bioimaging, Biosensing and Cancer Therapy. Chem. Commun..

[37] Xiao X., Chen M., Zhang Y., Li L., Peng Y., Li J., Zhou W. (2022). Hemin-Incorporating
DNA Nanozyme Enabling Catalytic
Oxygenation and GSH Depletion for Enhanced Photodynamic Therapy and
Synergistic Tumor Ferroptosis. J. Nanobiotechnol.

[38] Bai Y., Shang Q., Wu J., Zhang H., Liu C., Liu K. (2022). Supramolecular Self-Assemblies
with Self-Supplying H2O2 and Self-Consuming
GSH Property for Amplified Chemodynamic Therapy. ACS Appl. Mater. Interfaces.

[39] Qiu L., Wang J., Conceição M., Liu S., Yang M., Chen W., Long M., Cheng X., Wood M. J. A., Chen J. (2023). Tumor-Targeted Glycogen Nanoparticles
Loaded with Hemin and Glucose Oxidase to Promote Tumor Synergistic
Therapy. Int. J. Biol. Macromol..

[40] Li J., Wu H., Yan Y., Yuan T., Shu Y., Gao X., Zhang L., Li S., Ding S., Cheng W. (2021). Zippered G-Quadruplex/Hemin
Dnazyme: Exceptional Catalyst for Universal Bioanalytical Applications. Nucleic Acids Res..

[41] Cao Y., Ding P., Yang L., Li W., Luo Y., Wang J., Pei R. (2020). Investigation and Improvement of
Catalytic Activity of G-Quadruplex/Hemin DNAzymes Using Designed Terminal
G-Tetrads with Deoxyadenosine Caps. Chem. Sci..

[42] Li M., Yao B., Jing C., Chen H., Zhang Y., Zhou N. (2022). Engineering
a G-Quadruplex-Based Logic Gate Platform for Sensitive Assay of Dual
Biomarkers of Ovarian Cancer. Anal. Chim. Acta.

[43] Li C., Hu Y., Shi T., Dong K., Wu T. (2024). Label-Free Colorimetric
Detection Platform Based on Catalytic Hairpin Self-Assembly and G-Quadruplex/Hemin
DNAzyme for Comprehensive Biomarker Profiling. Talanta.

[44] Wang Y., Xiao J., Lin X., Waheed A., Ravikumar A., Zhang Z., Zou Y., Chen C. (2023). A Self-Assembled G-Quadruplex/Hemin
DNAzyme-Driven DNA Walker Strategy for Sensitive and Rapid Detection
of Lead Ions Based on Rolling Circle Amplification. Biosensors.

[45] Galli S., Melidis L., Flynn S. M., Varshney D., Simeone A., Spiegel J., Madden S. K., Tannahill D., Balasubramanian S. (2022). DNA G-Quadruplex Recognition In Vitro and in Live Cells
by a Structure-Specific Nanobody. J. Am. Chem.
Soc..

[46] Hu X., Zhang D., Zeng Z., Huang L., Lin X., Hong S. (2022). Aptamer-Based Probes for Cancer Diagnostics and Treatment. Life.

[47] Jiang S., Liu Q., Hu J., Yuan D., Zhang Y., Zhang C.-Y. (2022). Target-Triggered
Assembly of Functional G-Quadruplex DNAzyme Nanowires for Sensitive
Detection of MiRNA in Lung Tissues. Sensors
Actuators B Chem..

[48] Liu L. S., Leung H. M., Cai Y., Lo P. K. (2024). Recent Progress
in Stimuli-responsive DNA-based Logic Gates: Design, Working Principles
and Biological Applications. Smart Mol..

[49] Yu G., Chen X. (2019). Host-Guest Chemistry
in Supramolecular Theranostics. Theranostics.

[50] Wankar J., Kotla N. G., Gera S., Rasala S., Pandit A., Rochev Y. A. (2020). Recent Advances
in Host–Guest Self-Assembled
Cyclodextrin Carriers: Implications for Responsive Drug Delivery and
Biomedical Engineering. Adv. Funct. Mater..

[51] Tang M., Song Y., Lu Y.-L., Zhang Y.-M., Yu Z., Xu X., Liu Y. (2022). Cyclodextrin-Activated Porphyrin Photosensitization
for Boosting Self-Cleavable Drug Release. J.
Med. Chem..

[52] Bi X., Feng J., Feng X., Li D., Wang Y., Zhao S., Zhang L. (2025). APE1-Activated and NIR-II Photothermal-Enhanced
Chemodynamic Therapy Guided by Amplified Fluorescence Imaging. Anal. Chem..

[53] Yuan Y., Zhao H., Guo Y., Tang J., Liu C., Li L., Yao C., Yang D. (2020). A Programmable Hybrid DNA Nanogel
for Enhanced Photodynamic Therapy of Hypoxic Glioma. Trans. Tianjin Univ..

[54] Liu Z., Luan X., Lu Q., Qin S., Zeng F., Li Z., He B., Song Y. (2025). Reactive Oxygen
Species Responsive
Nanomotors for Gene Edited Metabolic Disruption and Immunotherapy. Nat. Commun..

[55] Zhang Y., Zhang K., Yang H., Hao Y., Zhang J., Zhao W., Zhang S., Ma S., Mao C. (2023). Highly Penetrable
Drug-Loaded Nanomotors for Photothermal-Enhanced Ferroptosis Treatment
of Tumor. ACS Appl. Mater. Interfaces.

[56] Sarkar A. R., Pal S., Sarkar A. K., Jana N. R. (2022). Hemin-Based Cell Therapy via Nanoparticle-Assisted
Uptake, Intracellular Reactive Oxygen Species Generation and Autophagy
Induction. New J. Chem..

[57] Zhang W., Fu Q., Luo Q., Sheng L., Yang J. (2021). Understanding Single-Atom
Catalysis in View of Theory. JACS Au.

[58] Han W., Wei Z., Feng L., Yao M., Zhang H., Zhang S. (2022). Single-Site
Fe-N-C Atom Based Carbon Nanotubes for Mutually Promoted and Synergistic
Oncotherapy. ACS Appl. Mater. Interfaces.

[59] Zhang L., Zhai B.-Z., Wu Y.-J., Wang Y. (2023). Recent Progress in
the Development of Nanomaterials Targeting Multiple Cancer Metabolic
Pathways: A Review of Mechanistic Approaches for Cancer Treatment. Drug Delivery.

[60] Lee D., Kwon S., Jang S., Park E., Lee Y., Koo H. (2022). Overcoming the Obstacles
of Current Photodynamic Therapy in Tumors
Using Nanoparticles. Bioact. Mater..

[61] Geng H., Ma L., Wu L., Yao C., Wang C., Gan X., Li Y., Chen F. (2025). Research on
the Function of GPX4 in Tumor-Targeted
Treatment Based on Its Molecular Structure and Features. Front. Oncol..

[62] Yu Q., Zhou J., Wang H., Liu Y., Zhou H., Kang B., Chen H.-Y., Xu J.-J. (2023). A Multiple-Response
Cascade Nanoreactor for Starvation and Deep Catalysis Chemodynamic
Assisted Near-Infrared-II Mild Photothermal Therapy. Chem. Biomed. Imaging.

[63] Li S., Wang Q., Jia Z., Da M., Zhao J., Yang R., Chen D. (2023). Recent Advances in
Glucose Oxidase-Based
Nanocarriers for Tumor Targeting Therapy. Heliyon.

[64] Liu P., Peng Y., Ding J., Zhou W. (2022). Fenton Metal Nanomedicines
for Imaging-Guided Combinatorial Chemodynamic Therapy against Cancer. Asian J. Pharm. Sci..

[65] Feng J., Chu C., Ma Z. (2021). Fenton and
Fenton-like Catalysts for Electrochemical
Immunoassay: A Mini Review. Electrochem. commun..

[66] Tong Z., Gao Y., Yang H., Wang W., Mao Z. (2021). Nanomaterials for Cascade
Promoted Catalytic Cancer Therapy. VIEW.

[67] Xing Z., Li L., Liao T., Wang J., Guo Y., Xu Z., Yu W., Kuang Y., Li C. (2024). A Multifunctional Cascade Enzyme
System for Enhanced Starvation/Chemodynamic Combination Therapy against
Hypoxic Tumors. J. Colloid Interface Sci..

[68] Ming J., Zhu T., Yang W., Shi Y., Huang D., Li J., Xiang S., Wang J., Chen X., Zheng N. (2020). Pd@Pt-GOx/HA
as a Novel Enzymatic Cascade Nanoreactor for High-Efficiency Starving-Enhanced
Chemodynamic Cancer Therapy. ACS Appl. Mater.
Interfaces.

[69] Ren H., Bai Y., Liu Z., Ma C., Tao X., Wang Q., Lian H., Li X. (2024). A Multifunctional Cascade Gas-Nanoreactor
with MnO2 as a Gatekeeper to Enhance Starvation Therapy and Provoke
Antitumor Immune Response. Acta Biomater..

[70] Zhang X., Zhang Y., Lv X., Zhang P., Xiao C., Chen X. (2024). DNA-Free Guanosine-Based
Polymer Nanoreactors with Multienzyme Activities
for Ferroptosis–Apoptosis Combined Antitumor Therapy. ACS Nano.

[71] Zheng H., Huang L., An G., Guo L., Wang N., Yang W., Zhu Y. (2024). A Nanoreactor Based
on Metal–Organic
Frameworks With Triple Synergistic Therapy for Hepatocellular Carcinoma. Adv. Healthcare Mater..

[72] Wang Y., Morrissey J. J., Gupta P., Chauhan P., Pachynski R. K., Harris P. K., Chaudhuri A., Singamaneni S. (2023). Preservation
of Proteins in Human Plasma through Metal–Organic Framework
Encapsulation. ACS Appl. Mater. Interfaces.

[73] Maeda Y., Kikuchi R., Kawagoe J., Tsuji T., Koyama N., Yamaguchi K., Nakamura H., Aoshiba K. (2020). Anti-Cancer Strategy
Targeting the Energy Metabolism of Tumor Cells Surviving a Low-Nutrient
Acidic Microenvironment. Mol. Metab..

[74] Xu Y., Ren M., Deng R., Meng J., Xu L., Zhao W., Ni Y., Mao C., Zhang S. (2025). UCNPs@PVP-Hemin-GOx@CaCO 3 Nanoplatform
for Ferroptosis Self-Amplification Combined with Calcium Overload. Adv. Healthc. Mater..

[75] Zhu X.-Y., Wang T.-Y., Jia H.-R., Wu S.-Y., Gao C.-Z., Li Y.-H., Zhang X., Shan B.-H., Wu F.-G. (2024). A Ferroptosis-Reinforced
Nanocatalyst Enhances Chemodynamic Therapy through Dual H2O2 Production
and Oxidative Stress Amplification. J. Controlled
Release.

[76] He X., Liu M., Du M., Huang Y., Xu P., Xie C., Fan Q., Zhou W. (2024). Self-Amplified Activatable Nanoprodrugs for Enhanced
Chemodynamic/Chemo Combination Therapy. Nanotechnology.

[77] Zhou H., Wang Z., Su Q., Qiu Q., Li J., Xu Z., Zhang M., Xiao J., Duan X. (2025). Self-Assembled Nanomedicine
Potentiates Tumor Ferroptosis. Chem. Eng. J..

[78] Su Y., Zhang Z., Lee L. T. O., Peng L., Lu L., He X., Zhang X. (2023). Amphiphilic
Dendrimer Doping Enhanced pH-Sensitivity
of Liposomal Vesicle for Effective Co-Delivery toward Synergistic
Ferroptosis–Apoptosis Therapy of Hepatocellular Carcinoma. Adv. Healthcare Mater..

[79] Li K., Xu K., He Y., Lu L., Mao Y., Gao P., Liu G., Wu J., Zhang Y., Xiang Y., Luo Z., Cai K. (2021). Functionalized
Tumor-Targeting Nanosheets Exhibiting Fe­(II) Overloading
and GSH Consumption for Ferroptosis Activation in Liver Tumor. Small.

[80] Feng K., Yoo E., Nie D., Houshyar Azar S., Cheng Z., Amirshaghaghi A., Tsourkas A. (2023). Hemin-Coated Nanoparticles as Drug Delivery Vehicles
for Chemotherapeutic Agents in Cancer Therapy. ACS Appl. Nano Mater..

[81] Xu C., Sun S., Johnson T., Qi R., Zhang S., Zhang J., Yang K. (2021). The Glutathione Peroxidase
Gpx4 Prevents Lipid Peroxidation and Ferroptosis
to Sustain Treg Cell Activation and Suppression of Antitumor Immunity. Cell Reports.

[82] Hu Q., Zhang Y., Lou H., Ou Z., Liu J., Duan W., Wang H., Ge Y., Min J., Wang F., Ju Z. (2021). GPX4 and Vitamin E Cooperatively
Protect Hematopoietic Stem and Progenitor Cells from Lipid Peroxidation
and Ferroptosis. Cell Death Dis.

[83] Yan Y., Teng H., Hang Q., Kondiparthi L., Lei G., Horbath A., Liu X., Mao C., Wu S., Zhuang L., James You M., Poyurovsky M. V., Ma L., Olszewski K., Gan B. (2023). SLC7A11 Expression Level Dictates
Differential Responses to Oxidative Stress in Cancer Cells. Nat. Commun..

[84] Sato M., Onuma K., Domon M., Hasegawa S., Suzuki A., Kusumi R., Hino R., Kakihara N., Kanda Y., Osaki M., Hamada J., Bannai S., Feederle R., Buday K., Angeli J. P. F., Proneth B., Conrad M., Okada F., Sato H. (2020). Loss of the Cystine/Glutamate
Antiporter
in Melanoma Abrogates Tumor Metastasis and Markedly Increases Survival
Rates of Mice. Int. J. Cancer.

[85] Xu Y., Xing Z., Abdalla
Ibrahim Suliman R., Liu Z., Tang F. (2024). Ferroptosis in Liver
Cancer: A Key Role of Post-Translational Modifications. Front. Immunol..

[86] Li F.-J., Long H.-Z., Zhou Z.-W., Luo H.-Y., Xu S.-G., Gao L.-C. (2022). System Xc–/GSH/GPX4 Axis:
An Important Antioxidant
System for the Ferroptosis in Drug-Resistant Solid Tumor Therapy. Front. Pharmacol..

[87] Tao J., Yuan Z., Zhou M. (2026). Recent Advances in Mitochondria-Targeted
Porphyrin-Based Metal-Organic Frameworks for Enhanced Cancer Therapy. Front. Pharmacol..

[88] Niu B., Liao K., Zhou Y., Wen T., Quan G., Pan X., Wu C. (2021). Application of Glutathione
Depletion in Cancer Therapy:
Enhanced ROS-Based Therapy, Ferroptosis, and Chemotherapy. Biomaterials.

[89] Chen M., Tong X., Sun Y., Dong C., Li C., Wang C., Zhang M., Wen Y., Ye P., Li R., Wan J., Liang S., Shi S. (2024). A Ferroptosis Amplifier
Based on Triple-Enhanced Lipid Peroxides Accumulation Strategy for
Effective Pancreatic Cancer Therapy. Biomaterials.

[90] Fu J., Liu M., Liu Y., Woodbury N. W., Yan H. (2012). Interenzyme Substrate
Diffusion for an Enzyme Cascade Organized on Spatially Addressable
DNA Nanostructures. J. Am. Chem. Soc..

[91] Chen J., Zhang Y., Cheng M., Mergny J.-L., Lin Q., Zhou J., Ju H. (2019). Highly Active
G-Quadruplex/Hemin
DNAzyme for Sensitive Colorimetric Determination of Lead­(II). Microchim. Acta.

[92] Larcher L. M., Pitout I. L., Keegan N. P., Veedu R. N., Fletcher S. (2023). DNAzymes:
Expanding the Potential of Nucleic Acid Therapeutics. Nucleic Acid Ther..

[93] Jiao Y., Shang Y., Li N., Ding B. (2022). DNA-Based Enzymatic
Systems and Their Applications. iScience.

[94] Hagiwara S., Momotake A., Ogura T., Yanagisawa S., Suzuki A., Neya S., Yamamoto Y. (2021). Effects of
Heme Electronic
Structure and Local Heme Environment on Catalytic Activity of a Peroxidase-Mimicking
Heme–DNAzyme. Inorg. Chem..

[95] Iwaniuk E. E., Adebayo T., Coleman S., Villaros C. G., Nesterova I. V. (2023). Activatable
G-Quadruplex Based Catalases for Signal Transduction in Biosensing. Nucleic Acids Res..

[96] Zuo L., Ren K., Guo X., Pokhrel P., Pokhrel B., Hossain M. A., Chen Z.-X., Mao H., Shen H. (2023). Amalgamation of DNAzymes
and Nanozymes in a Coronazyme. J. Am. Chem.
Soc..

[97] Kuche K., Yadav V., Patel M., Chaudhari D., Date T., Jain S. (2024). Enhancing Anti-Cancer Potential by
Delivering Synergistic Drug Combinations via Phenylboronic Acid Modified
PLGA Nanoparticles through Ferroptosis-Based Therapy. Biomater Adv..

[98] Mohaghegh N., Ahari A., Zehtabi F., Buttles C., Davani S., Hoang H., Tseng K., Zamanian B., Khosravi S., Daniali A., Kouchehbaghi N. H., Thomas I., Serati Nouri H., Khorsandi D., Abbasgholizadeh R., Akbari M., Patil R., Kang H., Jucaud V., Khademhosseini A., Hassani Najafabadi A. (2023). Injectable
Hydrogels for Personalized Cancer Immunotherapies. Acta Biomater..

[99] Chen J., Wang W., Wang Y., Yuan X., He C., Pei P., Su S., Zhao W., Luo S.-Z., Chen L. (2022). Self-Assembling Branched Amphiphilic Peptides for Targeted Delivery
of Small Molecule Anticancer Drugs. Eur. J.
Pharm. Biopharm..

[100] Yan Z., Bai Y., Zhang S., Kong L., Wang Y., Sun H., Li Y., Qiu L., Zhang R., Jiang P., Zhao D., Chen Z., Li Y., Pang H., Wang J. (2025). Quasi Fe MIL-53 Nanozyme Inducing
Ferroptosis and Immunogenic Cell
Death for Cancer Immunotherapy. Nat. Commun..

[101] An P., Hu D., Han Y., Meng H., Zhang X. (2022). Glucose Oxidase
Immobilization on Hemin@Pcn-222 (Mn): Integrated Biomimetic and Bioenzyme
Activities in Cascade Catalytic Process. SSRN
Electron. J..

[102] Wang J., Imaz I., Maspoch D. (2022). Metal–Organic
Frameworks: Why Make Them Small?. Small Struct..

[103] Hu C., Qu J., Xiao Y., Zhao S., Chen H., Dai L. (2019). Carbon Nanomaterials
for Energy and Biorelated Catalysis: Recent
Advances and Looking Forward. ACS Cent. Sci..

[104] Falara P. P., Zourou A., Kordatos K. V. (2022). Recent Advances
in Carbon Dots/2-D Hybrid Materials. Carbon
N. Y..

[105] Hasan M., Abrahamczyk S., Awan M. A., Sakreida O., Bachmatiuk A., Simha Martynková G., Čech
Barabaszová K., Rümmeli M. H. (2025). CVD-Engineered Nano Carbon Architectures:
Mechanisms, Challenges, and Outlook. Nanomaterials.

[106] Timilsina M. S., Zhu Z., Pandey R., Singh J., Ola O., Sahoo S., Hussain I., Tiwari S. K. (2026). Advancement in Nanocarbon-Based
Thermoelectric Materials: Surface Modification Strategies, Efficiency
Analysis, and Applications. J. Mater. Chem.
A.

[107] Xu C., Zhou L., Zheng S., Yu D., Tang Y., Fan C., Lin C., Zhou D., Lin Y. (2021). Hemin Covalently Functionalized
Carbon Nanobranch with Enzyme-Like and Photocatalytic Activities for
Synergistic Dye Degradation and Antibacterial Therapy. Adv. Sustain. Syst..

[108] Sugiyama M., Yurtsever A., Uenodan N., Nabae Y., Fukuma T., Hayamizu Y. (2025). Hierarchical
Assembly of Hemin-Peptide
Catalytic Systems on Graphite Surfaces. ACS
Nano.

[109] Jian T., Zhou Y., Wang P., Yang W., Mu P., Zhang X., Zhang X., Chen C.-L. (2022). Highly Stable and
Tunable Peptoid/Hemin Enzymatic Mimetics with Natural Peroxidase-like
Activities. Nat. Commun..

[110] Kumari S., Mandal S., Das P. (2019). Carbon Dot
Mediated
G Quadruplex Nano-Network Formation for Enhanced DNAzyme Activity
and Easy Catalyst Reclamation. RSC Adv..

[111] Villalba-Rodríguez A. M., Martínez-Zamudio L. Y., Martínez S. A. H., Rodríguez-Hernández J. A., Melchor-Martínez E. M., Flores-Contreras E. A., González-González R. B., Parra-Saldívar R. (2023). Nanomaterial
Constructs for Catalytic Applications in Biomedicine: Nanobiocatalysts
and Nanozymes. Top. Catal..

[112] Mohammadi E., Zeinali M., Mohammadi-Sardoo M., Iranpour M., Behnam B., Mandegary A. (2020). The Effects
of Functionalization of Carbon Nanotubes on Toxicological Parameters
in Mice. Hum Exp Toxicol.

[113] Liao J., Yao Y., Lee C.-H., Wu Y., Li P. (2021). In Vivo Biodistribution, Clearance, and Biocompatibility
of Multiple
Carbon Dots Containing Nanoparticles for Biomedical Application. Pharmaceutics.

[114] Mitev D. P., Alsharabasy A. M., Morrison L., Wittig S., Diener C., Pandit A. (2021). Plasma & Microwaves as Greener
Options for Nanodiamond Purification: Insight into Cytocompatibility. Front. Bioeng. Biotechnol..

[115] Pandit A., Alsharabasy A., Warneke J., Warneke Z., Glynn S., Farràs P. (2022). Model Hemin Derivatives as a New
Generation of Iron-Based Nitric Oxide Scavengers. ChemRxiv.

[116] Alsharabasy A. M., Glynn S., Farràs P., Pandit A. (2022). Protein Nitration Induced by Hemin/NO: A Complementary
Mechanism through the Catalytic Functions of Hemin and NO-Scavenging. Nitric Oxide.

[117] Jiang B., Guo Z., Liang M. (2023). Recent Progress in
Single-Atom Nanozymes Research. Nano Res..

[118] Obaid Saleh R., Saleh E. A. M., Moharam M. M., Uthirapathy S., Ballal S., Singh A., Nanda A., Ray S., Nasir A., Kaurshead R. S. (2025). Recent Trends and Advances in Single-Atom
Nanozymes for the Electrochemical and Optical Sensing of Pesticide
Residues in Food and Water. RSC Adv..

[119] Li T., Wang X., Wang Y., Zhang Y., Li S., Liu W., Liu S., Liu Y., Xing H., Otake K., Kitagawa S., Wu J., Dong H., Wei H. (2024). Microenvironmental
Modulation Breaks Intrinsic pH Limitations of Nanozymes to Boost Their
Activities. Nat. Commun..

[120] Zhang R., Xue B., Tao Y., Zhao H., Zhang Z., Wang X., Zhou X., Jiang B., Yang Z., Yan X., Fan K. (2022). Edge-Site
Engineering
of Defective Fe–N4 Nanozymes with Boosted Catalase-Like Performance
for Retinal Vasculopathies. Adv. Mater..

[121] Koebke K. J., Kühl T., Lojou E., Demeler B., Schoepp-Cothenet B., Iranzo O., Pecoraro V. L., Ivancich A. (2021). The pH-Induced
Selectivity Between Cysteine or Histidine Coordinated Heme in an Artificial
α-Helical Metalloprotein. Angew. Chem.,
Int. Ed..

[122] Srinivas U. S., Tan B. W. Q., Vellayappan B. A., Jeyasekharan A. D. (2019). ROS and the DNA Damage Response in Cancer. Redox Biol..

[123] Zhang H., Cui P., Xie D., Wang Y., Wang P., Sheng G. (2023). Axial N Ligand-Modulated Ultrahigh
Activity and Selectivity Hyperoxide Activation over Single-Atoms Nanozymes. Adv. Sci..

[124] Guo Z., Hong J., Song N., Liang M. (2024). Single-Atom Nanozymes:
From Precisely Engineering to Extensive Applications. Accounts Mater. Res..

[125] Li X., Xiang Z. (2022). Identifying the Impact
of the Covalent-Bonded Carbon
Matrix to FeN4 Sites for Acidic Oxygen Reduction. Nat. Commun..

[126] Wang J., Gao Y., Chen F., Zhang L., Li H., de Rooij N. F., Umar A., Lee Y.-K., French P. J., Yang B., Wang Y., Zhou G. (2022). Assembly of Core/Shell
Nanospheres of Amorphous Hemin/Acetone-Derived Carbonized Polymer
with Graphene Nanosheets for Room-Temperature NO Sensing. ACS Appl. Mater. Interfaces.

[127] Lu Y., Li W., Fan Y., Cheng L., Tang Y., Sun H. (2024). Recent Advances in
Bonding Regulation of Metalloporphyrin-Modified
Carbon-Based Catalysts for Accelerating Energy Electrocatalytic Applications. Small.

[128] Cao X., Zhu C., Hong Q., Chen X., Wang K., Shen Y., Liu S., Zhang Y. (2023). Insight into Iron Leaching
from an Ascorbate-Oxidase-like Fe–N–C Nanozyme and Oxygen
Reduction Selectivity. Angew. Chem..

[129] Yin Y., Ge X., Ouyang J., Na N. (2024). Tumor-Activated in
Situ Synthesis of Single-Atom Catalysts for O2-Independent Photodynamic
Therapy Based on Water-Splitting. Nat. Commun..

[130] Xue H., Li Y., Ma X., Zhang Q., Zhu J., Ni X., Qiu J., Li Z., Mu Z. (2025). Enhancing the Antioxidant
Capacity of Fe Single-Atom Nanozymes through Local Coordination Manipulation
for Psoriasis Treatment. Mater. Today Bio.

[131] Li R., Qiao X., Ma H., Li H., Li C., Jin L. (2022). In Situ Generated Fe3C Embedded Fe–N-Doped
Carbon Nanozymes
with Enhanced Oxidase Mimic Activity for Total Antioxidant Capacity
Assessment. J. Mater. Chem. B.

[132] Xiong Y., Huang J., Wang S.-T., Zafar S., Gang O. (2020). Local Environment
Affects the Activity of Enzymes on a 3D Molecular
Scaffold. ACS Nano.

[133] Cañeque T., Baron L., Müller S., Carmona A., Colombeau L., Versini A., Solier S., Gaillet C., Sindikubwabo F., Sampaio J. L., Sabatier M., Mishima E., Picard-Bernes A., Syx L., Servant N., Lombard B., Loew D., Zheng J., Proneth B., Thoidingjam L. K., Grimaud L., Fraser C. S., Szylo K. J., Der Kazarian E., Bonnet C., Charafe-Jauffret E., Ginestier C., Santofimia-Castaño P., Estaras M., Dusetti N., Iovanna J. L., Cunha A. S., Pittau G., Hammel P., Tzanis D., Bonvalot S., Watson S., Gandon V., Upadhyay A., Pratt D. A., Freitas F. P., Friedmann Angeli J. P., Stockwell B. R., Conrad M., Ubellacker J. M., Rodriguez R. (2025). Activation of Lysosomal Iron Triggers Ferroptosis in
Cancer. Nature.

[134] Vázquez-Meza H., Vilchis-Landeros M. M., Vázquez-Carrada M., Uribe-Ramírez D., Matuz-Mares D. (2023). Cellular Compartmentalization, Glutathione
Transport and Its Relevance in Some Pathologies. Antioxidants.

[135] Di Bona M., Chen Y., Agustinus A. S., Mazzagatti A., Duran M. A., Deyell M., Bronder D., Hickling J., Hong C., Scipioni L., Tedeschi G., Martin S., Li J., Ruzgaitė A., Riaz N., Shah P., D’Souza E. K., Brodtman D. Z., Sidoli S., Diplas B., Jalan M., Lee N. Y., Ordureau A., Izar B., Laughney A. M., Powell S., Gratton E., Santaguida S., Maciejowski J., Ly P., Jeitner T. M., Bakhoum S. F. (2024). Micronuclear
Collapse from Oxidative Damage. Science (80)..

[136] Zhang Y., Liao J., Liang H. (2023). MOF-Based G–Quadruplex/Hemin
DNAzymes for Cascade Reaction. Catalysts.

[137] Wang Y., Xie F., Zhao L. (2024). Spatially
Confined
Nanoreactors Designed for Biological Applications. Small.

[138] Somerville S. V., O’Mara P. B., Benedetti T. M., Cheong S., Schuhmann W., Tilley R. D., Gooding J. J. (2023). Nanoconfinement
Allows a Less Active Cascade Catalyst to Produce More C 2+ Products
in Electrochemical CO2 Reduction. J. Phys. Chem.
C.

[139] Jiang Z., Li J., Liu G., Qiu Q., Zhang J., Hao M., Ren H., Zhang Y. (2024). A pH-Sensitive
Glucose Oxidase and Hemin Coordination Micelle for Multi-Enzyme Cascade
and Amplified Cancer Chemodynamic Therapy. Small.

[140] Taneva S. G., Krumova S., Bogár F., Kincses A., Stoichev S., Todinova S., Danailova A., Horváth J., Násztor Z., Kelemen L., Dér A. (2021). Insights into
Graphene Oxide Interaction with Human Serum Albumin in Isolated State
and in Blood Plasma. Int. J. Biol. Macromol..

[141] Côa F., Delite F. de S., Strauss M., Martinez D. S. T. (2022). Toxicity
Mitigation and Biodistribution of Albumin Corona Coated Graphene Oxide
and Carbon Nanotubes in *Caenorhabditis Elegans*. NanoImpact.

[142] Song H., Zhang M., Tong W. (2022). Single-Atom Nanozymes:
Fabrication, Characterization, Surface Modification and Applications
of ROS Scavenging and Antibacterial. Molecules.

[143] Zhang H., Zhang S., Zhang Z. (2024). Advancements
and Applications
of Single-Atom Nanozymes in Sensing Analysis. Chemosensors.

[144] Khan S., Burciu B., Filipe C. D. M., Li Y., Dellinger K., Didar T. F. (2021). DNAzyme-Based Biosensors: Immobilization
Strategies, Applications, and Future Prospective. ACS Nano.

[145] Qiu D., He F., Liu Y., Zhou Z., Yang Y., Long Z., Chen Q., Chen D., Wei S., Mao X., Zhang X., Mergny J., Monchaud D., Ju H., Zhou J. (2024). A Cost-Effective Hemin-Based Artificial Enzyme Allows for Practical
Applications. Adv. Sci..

